# Foundations of plasma standards

**DOI:** 10.1088/1361-6595/acb810

**Published:** 2023-02-21

**Authors:** Luís L Alves, Markus M Becker, Jan van Dijk, Timo Gans, David B Go, Katharina Stapelmann, Jonathan Tennyson, Miles M Turner, Mark J Kushner

**Affiliations:** 1 Instituto de Plasmas e Fusão Nuclear, Instituto Superior Técnico, Universidade de Lisboa, Lisbon, Portugal; 2 Leibniz Institute for Plasma Science and Technology (INP), Greifswald, Germany; 3 Department of Applied Physics, Eindhoven University of Technology, Eindhoven, The Netherlands; 4 School of Physical Sciences and National Centre for Plasma Science and Technology, Faculty of Science and Health, Dublin City University, Dublin, Ireland; 5 Department of Aerospace and Mechanical Engineering, University of Notre Dame, Notre Dame, IN 46556-5637, United States of America; 6 Department of Nuclear Engineering, North Carolina State University, Raleigh, NC 27695, United States of America; 7 Department of Physics and Astronomy, University College London, London, United Kingdom; 8 Electrical Engineering and Computer Science Department, University of Michigan, Ann Arbor, MI, 48109-2122, United States of America

**Keywords:** standard plasma sources, plasma diagnostics, standards and best practice, data and reaction mechanisms, verification and validation, open source codes, plasma dose

## Abstract

The field of low-temperature plasmas (LTPs) excels by virtue of its broad intellectual diversity, interdisciplinarity and range of applications. This great diversity also challenges researchers in communicating the outcomes of their investigations, as common practices and expectations for reporting vary widely in the many disciplines that either fall under the LTP umbrella or interact closely with LTP topics. These challenges encompass comparing measurements made in different laboratories, exchanging and sharing computer models, enabling reproducibility in experiments and computations using traceable and transparent methods and data, establishing metrics for reliability, and in translating fundamental findings to practice. In this paper, we address these challenges from the perspective of LTP standards for measurements, diagnostics, computations, reporting and plasma sources. This discussion on standards, or recommended best practices, and in some cases suggestions for standards or best practices, has the goal of improving communication, reproducibility and transparency within the LTP field and fields allied with LTPs. This discussion also acknowledges that standards and best practices, either recommended or at some point enforced, are ultimately a matter of judgment. These standards and recommended practices should not limit innovation nor prevent research breakthroughs from having real-time impact. Ultimately, the goal of our research community is to advance the entire LTP field and the many applications it touches through a shared set of expectations.

## 1. Introduction

In the realm of research and development in the field of low-temperature plasmas (LTPs), there is a natural transition or evolution from concept to implementation. An embryonic idea or concept is first investigated with there being little understanding of the underlying processes. Measurements are difficult to perform in this new parameter space, and models are difficult to formulate in the absence of fundamental data and knowing the dominant processes (and how to computationally represent them). Experimental data and model results both have large uncertainty bars. In spite of these outcomes not having high precision, the *first-time* trends revealed by these perhaps not precise results provide remarkable insights to new processes or a better understanding of the underlying physics. They stimulate thought and innovation. As a result of these works, it becomes more clear what data are required to make more precise measurements and build more focused models. Through this improvement in understanding, more definitive experiments are designed and conducted, more relevant data is produced and more representative models are constructed. Uncertainty bars are reduced and understanding is improved. The precision of the investigation and of our understanding is improved.

This cycle of improved understanding enabling more definitive experiments in turn enables precision to be improved. When there is sufficient fundamental understanding of the basic underlying processes, then attention expands to address higher levels of precision. This higher level of precision produces refined reaction mechanisms and data, and smaller linewidths in spectroscopic measurements. This precision then enables yet higher levels of precision. When understanding and precision reach a threshold level, technology transfer begins wherein, for example, plasma sources are designed and built to provide reproducible and predictable *doses* of plasma activated species. The end result is often a commercial product.

Underlying this transition in concept to implementation, and increasing degrees of decision, should be a culture of standards. A dictionary definition of standards is ‘something set up and established by authority as a rule for the measure of quantity, weight, extent, value, or quality’ [1]. Standards are absolutely critical and necessary in applications involving life-safety, and are embodied in building codes and crash-worthiness requirements for automobiles. Another perspective of standards is ‘an established norm or requirement for a repeatable technical task which is applied to a common and repeated use of rules, conditions [and] guidelines…’ [2]. This perspective emphasizes the need for common practices to enable exchange of information and to gauge the goodness of a process.

The field of LTPs extends from concept to commercialization, and so has an extremely diverse need for standards enabling increasing levels of precision, with different levels of expectation for compliance. One class of standards is, in principle, independent of where one lies in the continuum between concept and precision. These standards address the exchange of information and data consistently, unambiguously and reproducibly; verifying a computer model, calibrating a diagnostic, or reporting on a result. Another class of standards applies when moving beyond conceptualizing, a realm in which higher levels of precision are possible. These standards address validation of codes, confirmation of reaction mechanisms, production and dissemination of data, and development of standard sources to enable measurements to be collated across laboratories. A final class of standards apply to the technology transfer end of the innovation chain. These standards address reproducibility, safety and reliability.

The proper and measured use of standards is critical to fostering the entire breadth of the innovation chain, from ideation to commercialization. Some standards should be thought of as being universal. These standards address transparency in reporting, defining techniques and methods, and making data available. As one transitions from concept to precision, the enforcement of standards becomes more appropriate. Premature enforcement of standards runs the risk of stifling innovation. Neglecting standards can lead to inefficiency and questionable conclusions.

In this paper, we discuss the development and use of standards in the LTP community. The term standards is used here in the most general way and is synonymous with recommended best practices and processes. The intent of the discussion in this paper is to provide guidance for how research results can be communicated and utilized by the LTP community more efficiently, more reliably, more reproducibly and less ambiguously. Achieving these goals works toward transparency, improving the acceptance of the research and accelerating advances in the field while not stifling innovation.

This paper has the following sections addressing the wide range of experimental, computational, theoretical and technology transfer elements of the LTP field. With the discussion in each section being wide ranging, here we also provide summary statements, *action-items* or *recommended path* for each section. These items are intended to provide the reader with a high level perspective of our recommendations.

### Plasma sources (Section 2): *A discussion of how standard plasma sources may benefit exchange of data and experiences between laboratories*.

While it is recognized that developing and deploying community-driven standard plasma sources is a large undertaking, experience with the gaseous electronics conference (GEC) reference cell (GECRC) and the Cooperation in Science and Technology (COST) Jet has shown how valuable standard sources can be. The community should, whenever possible, use the already existing standard sources as ‘calibration tools’ for their own plasma devices to improve fundamental understanding of plasma processes and accelerate translation of scientific findings to applications. Further standards that are easier to realize than a complex standard plasma source, for example a standard substrate to investigate plasma-surface interactions, should be the next focus of community-driven standardization efforts.

### Plasma diagnostics (Section 3): *In developing and reporting on measurements, what information and practices are required to ensure transparency and reproducibility?*

In the absence of established diagnostics standards, it is recommended that in their reporting, researchers follow a minimum framework to support transparency. This framework should include: a clear and detailed description of the equipment used for the diagnostics, the precise implementation of the diagnostics, the input data to the analysis and the analysis techniques as well as access to the raw data. This would allow potential reanalyzing of the data using different techniques or when analysis techniques are improved.

### Data and reaction mechanisms (Section 4): *Diagnostics and modeling require fundamental data and reaction mechanisms. How should these data and mechanisms be distributed, archived and validated?*

Data standards in LTP physics have been developed in a meritocratic way where individuals or organizations propose standards. The standards that succeed are those that bring the most benefits to the community. These successes heavily depend on the availability and usability of tools that can utilize the data. A good example of such a successful combination is the LXCat database and the BOLSIG+ program for solving Boltzmann’s equation. This meritocratic process will likely continue for some time. Wider adoption of standards than has happened to date will depend on the willingness of the standards developers to listen better to the needs and wishes of the community, and to look beyond the boundaries of their respective application domains.

### (Quick) Data generation (Section 5): *In the absence of experiments or first principles calculations, are there standard methods to quickly produce needed data for models and diagnostics?*

The input data, regardless of the method of generation, need proper statements of uncertainties. Procedures for uncertainty quantification, particularly for theoretical/calculated data, need to be developed and routinely applied. To speed the generation of this needed data, machine learning should be explored as a route to providing data on the many processes for which little or nothing is now known.

### Verification and validation V&V (Section 6): *How can standards work toward improving the reliability and accuracy of computer models?*

In the short term, authors should ensure that their reporting of calculations fully meets the requirements of scientific reproducibility, by making certain that the algorithms, numerical parameters and data that have been employed are fully specified and traceable (in the case of data). Whenever practical, source code and data files should be made available. Editors and referees should consider carefully what minimal standards are appropriate in their context, taking note of the considerations discussed above.

### Open source and publicly available codes (Section 7): *In development and maintenance of computer models, how can standards improve access and speed innovation?*

The LTP community should use the main conferences in the field as a forum to design and carry out collective efforts for the verification and benchmarking of codes. These efforts could be directed toward open-source codes as a means to develop specific recommendations on revisions to the codes, as well as directed toward less available codes as a means to guide the authors of those codes.

### Reporting (Section 8): *Nearly every technical endeavor produces a report—a journal article or archival document. What are the expectations for transparency in reporting that aid in the communication and reproducibility of results?*

Reporting standards for the most part simply reflect proper actualization and effective communication of the scientific method. That is, proper reporting should necessarily be a central element of journal articles. Practically, editors and publishers of journals popular among the LTP community, including the one publishing this paper, could adopt submission checklists or similar mechanisms, not unlike Nature, that ensure that published papers follow expected reporting conventions.

### Plasma dose (Section 9): *Many fields have standards for quantifying reactivity. Can such a standard be developed for LTPs?*

The definition and use of dose (e.g., energy deposited to produce a given product) has been successfully adopted in fields such as radiation physics. Those successes result in part from the generation and delivery of the activating energy being separable from interaction with the target, and due to the dose itself being weakly sensitive to the absolute energy of delivery. The definition of dose for LTPs has been considerably less successful due to the lack of this separability and due to product generation being sensitive to the distribution of the delivered energy. Advancing the concept of dose for LTPs, useful for calibrating plasma sources and protocols, would benefit from having different categories of doses associated with, for example, reactant generation and use of those reactant fluxes. These definitions would best be refined by community led efforts.

### Technology transfer (Section 10): *One outcome of translational research is technology transfer and commercialization. What standards will enhance this process?*

To support technology transfer, data should be collected systematically and under well-defined conditions. The definition of reporting standards and the use of interoperable data formats will further help to overcome the hurdles in establishing new plasma applications.

## 2. Plasma sources

Standard plasma sources have the goal of providing *identical* experimental platforms for researchers in different laboratories. These identical platforms would enable a variety of experimental techniques to be employed and modeling performed with there being a minimum in laboratory-to-laboratory variation in reactor configuration and operating conditions. In this way, researchers can exchange and compare experimental data and modeling results with the goal of gaining fundamental understanding of the physical and chemical properties of the plasma and plasma chemistry. This would be accomplished without needing to correct or account for differences in these data due to known (or unknown) differences in the reactors or operating conditions. Community-driven efforts to use one experimental platform that can be deployed in various laboratories where researchers with different expertise contribute to the development of a ‘big picture’ have been successfully employed with the *GECRC* and later with the *COST reference microplasma jet*, both described in more detail below. Data generated from different research groups can help contextualize and cross correlate results, and to accelerate the understanding of fundamental processes.

### 2.1. Currently available standard plasma sources

#### 2.1.1. GECRC.

**Figure 1.**
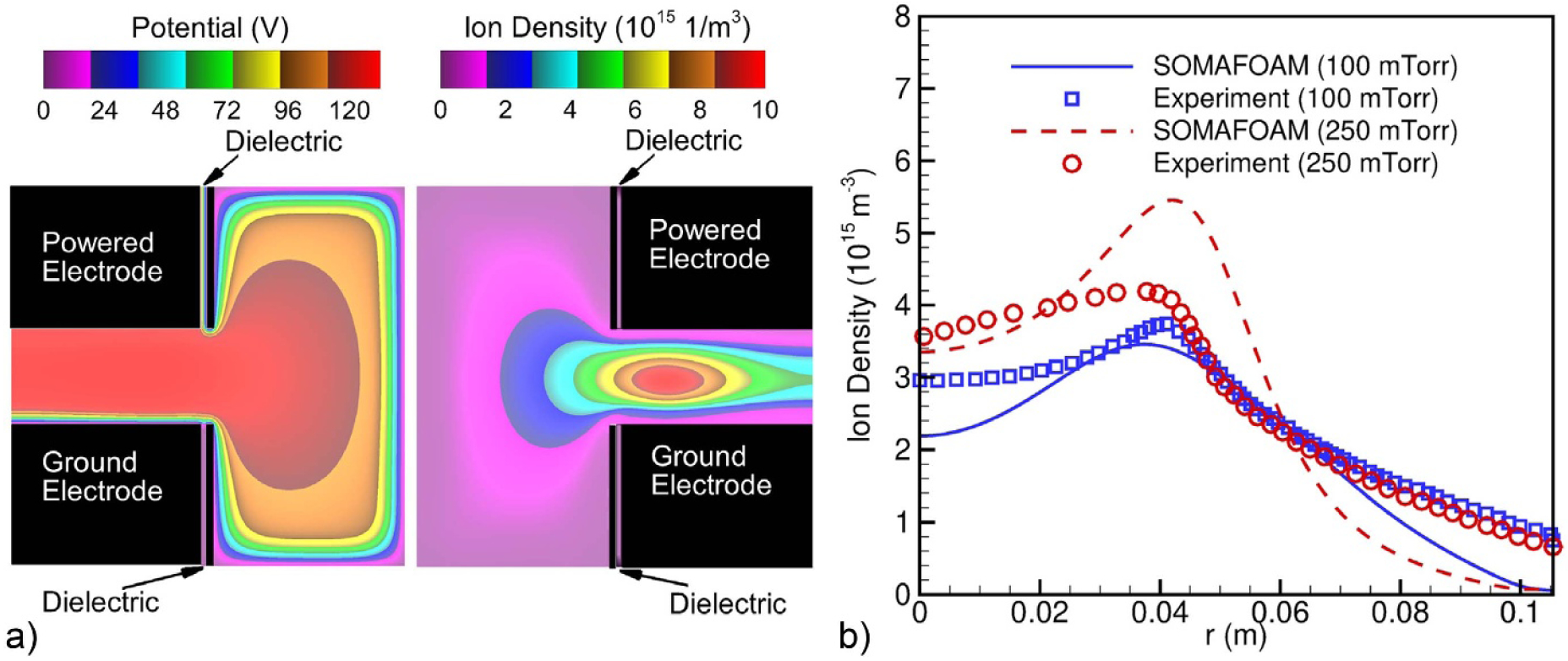
The GECRC continues to be a benchmark for validating codes and investigating fundamental plasma properties. (a) Simulation results using the SOMAFOAM platform for plasma potential and Ar^+^ density in a CCP sustained in 100 mTorr Ar powered with 400 V peak-to-peak at 13.56 MHz. (b) Comparison to experimental results. Reprinted from [6], Copyright (2021), with permission from Elsevier.

The concept of the GECRC grew out of a workshop field at the GEC in Minneapolis, Minnesota (USA) in 1988 and was further refined by the GEC community in the following year. (The GEC in 1989 was field in Palo Alto, California (USA) south of San Francisco. The ‘Workshop on the Reference System for RF Plasma Processing Research’ took place on 17 October, scheduled for 3:45 pm–5:30 pm. Just as Joseph T Verdeyen summarized his points at 5:04 pm, the Loma Prieta earthquake occurred, concluding the workshop with Professor Verdeyen having the final word.) The GECRC was originally designed to advance the understanding of radio frequency (RF) excited capacitively coupled plasmas (CCPs) of the type used in microelectronics fabrication [3] and later to address inductively coupled plasmas (ICPs) as those devices began to be used in the microelectronics industry. By having a standard cell that would be implemented in laboratories throughout the world, measurements made in different laboratories and modeling could be compared on a side-by-side basis (see example in figure 1). Eliminating lab-specific aspects of the plasma source (e.g. wall materials, distance from the powered electrodes to side walls, pumping scheme) enabled there to be more focus on the properties of the plasma. The GECRC was widely adopted with dozens of cells employed throughout the world, enabled in part by a commercial vacuum equipment company having a part-number for ordering an assembled cell at moderate cost. The GECRC is still actively used more than 30 years later, having served as a valuable asset to the research community [4, 5] and continues to be a benchmark for validating codes (see figure 1 as a recent example).

This initial foray into standard plasma sources was an eye-opening experience for all concerned. This eye-opening was a result of under-estimating the importance to measurements and plasma properties of what were considered minor differences in power-supplies, matching networks and electrical connections. After deployment of the first GECRC, a workshop was held to compare measurements of the most basic properties of a CCP sustained in argon—current, voltage, phase and electron density. There were hugely unexpected variations in even the current–voltage measurements made in different laboratories. These variations were eventually attributed to subtle and sometimes not so subtle differences between laboratories in the matching networks, how cables were configured between the power supplies, matchboxes and plasma chamber; and stray capacitance and inductance due to what were thought to be inconsequential modifications of the cell. This experience focused the entire community’s attention on viewing plasma sources as a system, beginning with the power supply, cables and matchboxes and extending through the plasma chamber and method of electrical termination. Guidelines were established for those components to achieve more reproducible results between laboratories. These experiences were translated to practice in the semiconductor plasma processing industry to help achieve reproducibility between plasma tools and in the same tools before and after maintenance. Although not appreciated at the time, gas impurities can also have a large impact on plasma properties (as discussed in section 3) and future reference plasma sources should include impurity specifications in at least their calibration procedures.

#### 2.1.2. COST reference microplasma jet.

**Figure 2.**
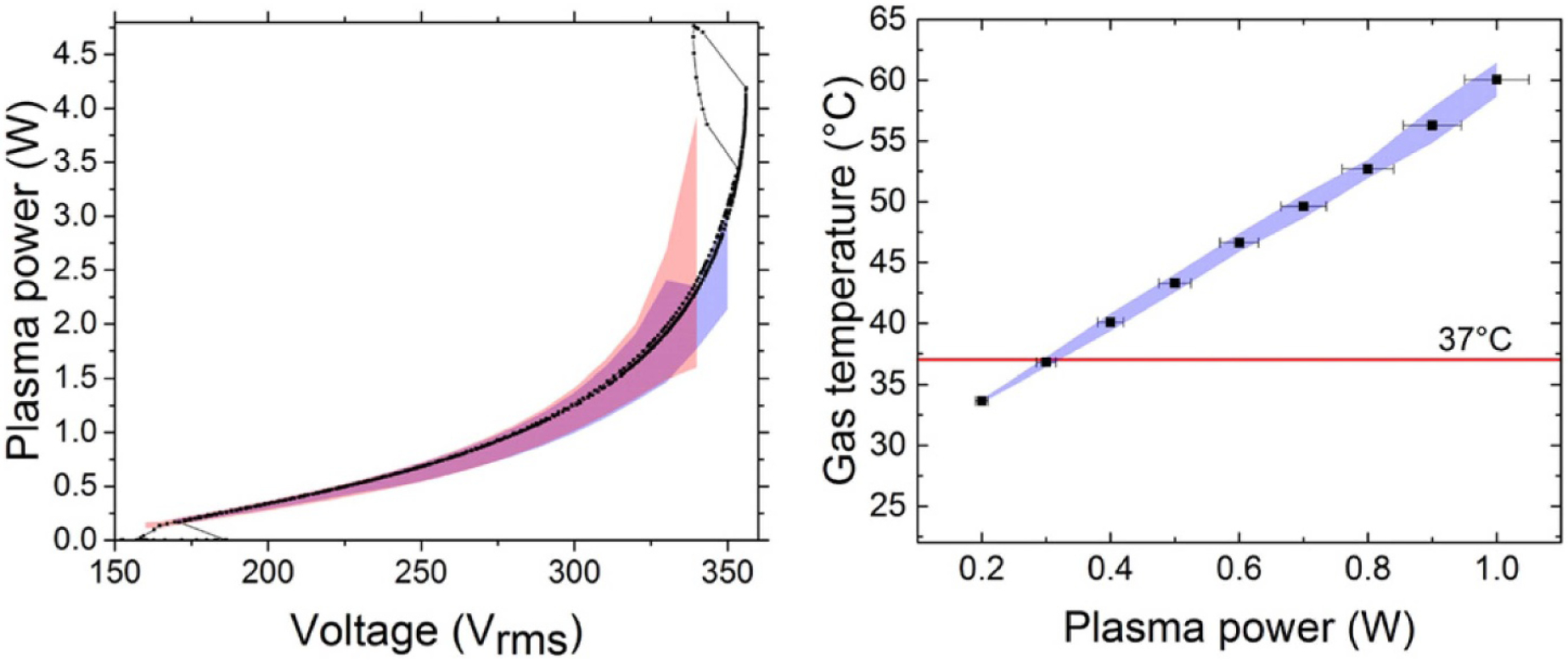
Reproducibility of the COST-jet: plasma and device properties in four sources were measured independently. The black dots indicate measured data from one jet, the shaded area indicates the deviation between the four sources. (Left) Plasma power as a function of voltage and (right) measured effluent gas temperature at 3 mm distance from the nozzle as a function of plasma power. Reproduced from [8]. © The Author(s). Published by IOP Publishing Ltd. CC BY 4.0.

The COST reference microplasma jet (COST-jet) originated through efforts supported by the European COST Action MP1011 on ‘Biomedical Applications of Atmospheric Pressure Plasma Technology’ [7]. Within this COST Action it was recognized that measurements of plasma properties made in a wide variety of mostly home-made atmospheric pressure plasma jets complicated comparison of results obtained in different laboratories, leading to delays in interpretation of those results and in furthering understanding of fundamental properties of the plasma. A working group was formed to define a device that could serve as a reference plasma jet for researchers in the sub-field of plasma medicine which relies heavily on plasma jets. The motivation to use a reference plasma source to advance understanding of processes occurring in atmospheric pressure plasma jets is similar to that of the GECRC. The end product was the COST-jet. Much effort has been devoted to improving the reproducibility of the COST-jet itself and among multiple devices in different laboratories [8]. See figure 2 as an example of these efforts.

The COST-jet has integrated voltage and current probes. A calibration protocol, instructions on how to measure and calculate dissipated power, and operation protocols for taking reproducible measurements have been published [7]. Several computational models to capture the chemical kinetics in the gas phase have been developed [9-11]. Applications of the COST-jet have been recently reviewed by Gorbanev *et al* [12] and range from polymer surface modifications [13] and studying anti-cancer effects [14], to investigating plasma-driven biocatalysis [15].

#### 2.1.3. Other commercial sources.

In addition to community-derived standard plasma sources such as the GECRC and COST-jet, commercial plasma sources are also available that could serve as standard sources. While these commercial sources were originally not intended to be research tools, they are manufactured to extreme precision and reproducibility. However, most commercial plasma sources are designed for application areas in which the plasma is delivered in a highly constrained manner (e.g. plasma etching reactors, hollow cathode lamps, and fluorescent lamps) with little access for diagnostics. Standard research plasma sources are intended to be reproducible with flexibility in operating conditions (pressure, power, gas mixture) and with access for diagnostics. In new application areas it is difficult to motivate commercial entities to offer standard sources or modify existing products, as the market is admittedly small, with there being an early success in doing so for the GECRC. That said, several commercial plasma sources have become de-facto standard sources due to their widespread use. For example, the commercial kINPen plasma jet has been extensively characterized and modeled [16], while being used in laboratories throughout the world.

The responsibility for building and maintaining community-driven standard sources may exceed the capability of the research community, which may explain that although successful, only two community-driven standard sources have been developed to date. The ideal situation may be a community-driven design that is transferred to a company which then commercializes the device, while also being open to making periodic community-driven modifications.

### 2.2. Opportunities and challenges for the LTP community

Standard plasma sources have the potential to advance the understanding of a particular type of plasma configuration and to accelerate research, including the translation of findings from basic research to industrial applications. Plasma medicine and plasma agriculture as well as plasma-based water purification have shown promising results over the last decade [17]. Many of the plasma devices used in these investigations have been under-diagnosed and operating conditions have been under-specified due to the emphasis being on the outcome of the application. For example, the electrical environment around plasma jets and the substrate are rarely mentioned in publications in spite of their having strong effects on the performance of the plasma apparatus. The end result has been a lack of reproducibility of the application focused results between laboratories and even within laboratories. This lack of reproducibility and lack of knowledge of fundamental properties, such as the flux of plasma generated radicals onto the sample, has resulted in limiting our understanding of the basic processes of the interaction of plasma with liquid or biological interfaces. Standard plasma sources in all of these areas would help to (a) contextualize results, (b) compare and benchmark experiments, and (c) develop and validate models for the plasmas.

The use of standard plasma sources should be a tool toward improving fundamental understanding of plasma processes and speeding translation of scientific findings to applications, while not hindering innovation. It would be detrimental to the field to mandate that to be credible, every researcher must use the same plasma sources, as it would also be detrimental to have no standard plasma sources. Comparing experimental and computational results obtained with standard plasma sources with other well characterized sources can facilitate broadening and extending the operational space. Doing so will similarly advance understanding and help to contextualize results and to identify mechanisms, as it has been done successfully with the COST-jet in comparison with the well-studied kINPen [16, 18, 19] and a ns-pulsed dielectric barrier discharge (DBD) [20]. In this mode of operation, standard plasma sources could act as ‘calibration sources’ for diagnostics and models, which may be particularly important in research areas using several reactor designs, developing new sources and scaling up processes.

The major emphasis to date on standard sources has been on plasma generation with there being less emphasis on plasma-surface interactions, in spite of the latter being a major outcome of researchers’ efforts. A community-driven design for a reference plasma-surface treatment system would enable combined fundamental investigations of the plasma source and interface behavior. The outcomes would directly assist in determining limitations and advantages of the process, performance levels in comparison to other plasma devices or existing technology, and to identify the key technical challenges that must be overcome before the technology can be upscaled.

In addition to standard plasma sources, standard protocols for making and calibrating measurements would help the community further understand basic processes in the plasma as well as in interfacial interactions. While standard protocols are common in other research areas, the LTP community lacks standard diagnostics and reporting formats, both further discussed in sections 3 and 8, respectively. The LTP community is extremely intellectually diverse, with a large variety of plasma devices and applications as well as a large variety of educational and training backgrounds. Diagnostics are often unique to a single laboratory and part of the research program itself (e.g. mass spectrometry for atmospheric pressure plasmas [21]) or widely used but without a standard procedure on how to calibrate and report results (e.g. optical emission spectroscopy (OES) [22, 23]). The end result has been difficulties in assembling results from multiple laboratories to create a more complete picture of plasma phenomena.

The LTP community is not the only research community facing challenges like this and several publishers recently launched protocol journals to work toward reproducibility by sharing a detailed, step-by-step protocol and to take advantage of expert peer review to refine and shape protocols. The LTP community can particularly benefit from these efforts as plasmas can be very sensitive to small variations in parameters or protocols due to the underlying complex and nonlinear physics. The LTP community is encouraged to develop and share standard protocols to increase the reproducibility between laboratories and to transfer knowledge. That said, the goal of having protocols is to improve reproducibility and sharing of results, and not to stifle innovation. It is sometimes a first-time measurement using a newly developed diagnostic on a new source that opens up an entirely new field of research. For example, the electric field induced second harmonic generation technique for measuring electric fields was demonstrated using a non-standard, home-built setup, and has since dramatically beneficially affected plasma diagnostics over the last 5 years since that demonstration [24, 25].

## 3. Plasma diagnostics

LTPs are weakly ionized complex multi-species systems, including charged species (electrons, positive ions, negative ions), ground state neutral species (gas-phase background species, dissociation products, reaction products), excited species (electronic, ro-vibrational) and photons. The different species are not in thermal equilibrium with each other and can exhibit very different temperatures and energy distributions. Light electrons typically have mean energies in the range of 1–5 eV while heavier species in the plasma bulk typically have temperatures only slightly elevated above room temperature, around hundreds of degrees Kelvin. Ions adjacent to surfaces that are accelerated in the boundary sheath electric field can reach energies of hundreds of eV. The densities of and dynamics of charged species transport are closely coupled with the spatial and temporal structure of the electric field, both local and remote.

The investigation and characterization of LTPs are often challenging and require the combination of several specialized diagnostic techniques for the measurement of species densities, energy distributions (temperatures) and electric fields. Frequently employed techniques include electrical diagnostics, mass spectrometry and optical diagnostics. The different techniques provide complementary information about various plasma parameters and each technique exhibits their own specific advantages and disadvantages depending on the plasma environment and parameters of interest. The role of diagnostics tends to be multifold, going beyond diagnosing and characterizing plasmas. Diagnostics also play key roles in validation of models and simulations as well as being sensors for process control in industrial settings.

Of paramount importance in deploying diagnostics is their reliability or accuracy. The assessment of reliability or accuracy is best made by comparing results of diagnostics to known, calibrated standard sources, or to a theoretical expression or computation if the conditions are amenable to theory. In the absence of standard sources or an applicable theory or computation, the reliability of diagnostics is often determined by whether they reproduce the measurements of other diagnostics. In these comparisons, the average value of a plasma quantity obtained from many measurements made in different laboratories is basically declared to be the ‘correct’ value. Outliers (least consistent with other measurements, e.g. outside a certain confidence band) are then considered to be ‘less correct’. As unsatisfying as this technique is, in the absence of a known, calibrated standard, applicable theory or computation, there are few other options. The liability of this method is that the outlier may indeed be the correct measurement.

### 3.1. Brief overview of frequently employed plasma diagnostics

A detailed discussion of various LTP diagnostics can be found in [26]. Only a brief overview of frequently employed techniques is outlined here, along with specific advantages and disadvantages.

Comparatively simple external electrical measurements of voltage and current are mostly non-intrusive and can provide useful information about charged species and fields in the plasma [27]. However, these data are indirect and require interpretation based on model assumptions for the plasma. Internal electrical probes [28], such as Langmuir probes, hairpin probes and retarding field analyzers, can provide more direct and spatially resolved information. Nevertheless, the analysis still requires model assumptions. An inherent disadvantage of internal electrical probes is the potentially intrusive nature and limited applicability in the harsh environments of reactive plasmas as well as thermal limitations of probe materials.

Mass spectrometry is typically an external non-intrusive technique to measure neutral particle and ion densities as well as energy distribution functions [29]. These are particularly valuable as they are key parameters in plasma-surface interactions. Details of the equipment and data interpretation can be complicated. Similar to internal electrical probes, harsh environments can also be a challenge for mass spectrometry due to its direct contact with reactive plasma species. That contact may change the composition of the measured species as well as the dissociation induced by the ionizer requiring the analysis of cracking patterns to identify complex species.

Optical diagnostics are versatile and can provide non-intrusive information about plasma parameters with high temporal and spatial resolution [30], e.g. through using intensified charge-coupled device (ICCD) imaging [31] or three dimensional computer assisted tomography [32]. Passive OES is experimentally comparatively simple and is truly non-obtrusive with the possible exception of modifying the plasma cell to provide optical access. Due to its robustness and comparatively low cost, optical emission is often employed for plasma monitoring. However, optical emission provides indirect information about plasma parameters. The raw optical emission data must be deconvolved to obtain absolute data (such as densities and temperatures) using what are sometimes complex model assumptions. Optical emission also strongly relies on the availability and accuracy of atomic and molecular data for, for example, oscillator strengths and collisional quenching coefficients.

Active optical diagnostics, often laser-based spectroscopy techniques, can provide direct and highly accurate information, typically only requiring moderate model assumptions [33]. Widely used techniques include absorption spectroscopy and laser induced fluorescence (LIF) spectroscopy. Absorption spectroscopy directly measures line-integrated absolute species densities through photon absorption using wavelengths ranging throughout the spectrum of vacuum ultraviolet (VUV), ultraviolet, visible and infrared. Light sources vary from synchrotron radiation and a broad variety of laser systems to comparatively simple classical light sources as, for example, commonly employed in Fourier transform infrared spectroscopy. Measurement sensitivity is often improved through multipass absorption techniques, either using simple multipass cells or cavity based approaches as in cavity ring down spectroscopy. LIF spectroscopy is also based on photon absorption [34]. This technique can encompass either one photon (LIF) or two-photon absorption LIF (TALIF) spectroscopy. In both techniques, LIF and TALIF, the subsequently emitted fluorescence photon is detected and analyzed. These techniques can provide high spatial and temporal resolution for accurate measurements of species densities. The measurements are valuable as reliable reference points for other diagnostic techniques as well as theoretical and computational investigations. However, they are experimentally involved and tend to require costly equipment.

It should be noted that all active optical diagnostics are intrusive (or perturbative) because the diagnostics change the density of the species being observed in a given atomic or molecular level. Even elastic scattering techniques (e.g. Rayleigh or Thomson scattering) can transfer power to the plasma. The degree of intrusiveness ranges from negligible to significant (the latter being the origin of diagnostic techniques such as optogalvanic spectroscopy [35]). It is the researchers’ responsibility to determine how perturbing their diagnostic is, and how to account for those perturbations in the analysis of the data.

### 3.2. Case study for diagnostics benchmark and simulation validation

In this subsection an example case study is discussed to illustrate benchmarking different diagnostic techniques against each other, and the interplay with computational simulations for validation. The case study focuses on plasma sources similar to the COST-jet discussed in section 2. The sources include slight modifications due to these studies being performed during different stages of development of the reference source, as well as there being modifications made to accommodate specific diagnostic requirements. The plasma sources operated in a helium gas flow through a 1 mm discharge gap with varying humidity admixtures. The power delivery was RF capacitively coupled at 13.56 MHz. Details of the individual experimental setups can be found in [10, 36, 37].

The diagnostics measured absolute atomic oxygen densities using different techniques: ns-TALIF [36], synchrotron VUV absorption [10] and picosecond TALIF (ps-TALIF) [37]. Synchrotron VUV absorption measurements are the most direct method using a one-photon absorption process having a well characterized absorption cross section. As such, the method does not require an additional calibration process. On the other hand, VUV absorption is also an experimentally demanding technique and limited to strict vacuum protocols, while providing a column density as opposed to a local density. Details of the VUV absorption process are discussed in [37, 38]. While still complex, ns-TALIF is experimentally the least demanding technique among the techniques that were compared. Nonetheless, ns-TALIF relies on estimates of collisional de-excitation rates and requires calibration, producing additional uncertainty. Details are discussed in [36, 37]. ps-TALIF reduces the need to estimate collisional de-excitation rates as typically required in ns-TALIF. With its shorter pulse length, direct measurements of effective de-excitation rates can be made. Similar to ns-TALIF, ps-TALIF still requires a calibration procedure, but can also be applied in more application relevant laboratory conditions. Nevertheless, ps-TALIF is also experimentally complex and costly to implement. Details are discussed in [37].

**Figure 3.**
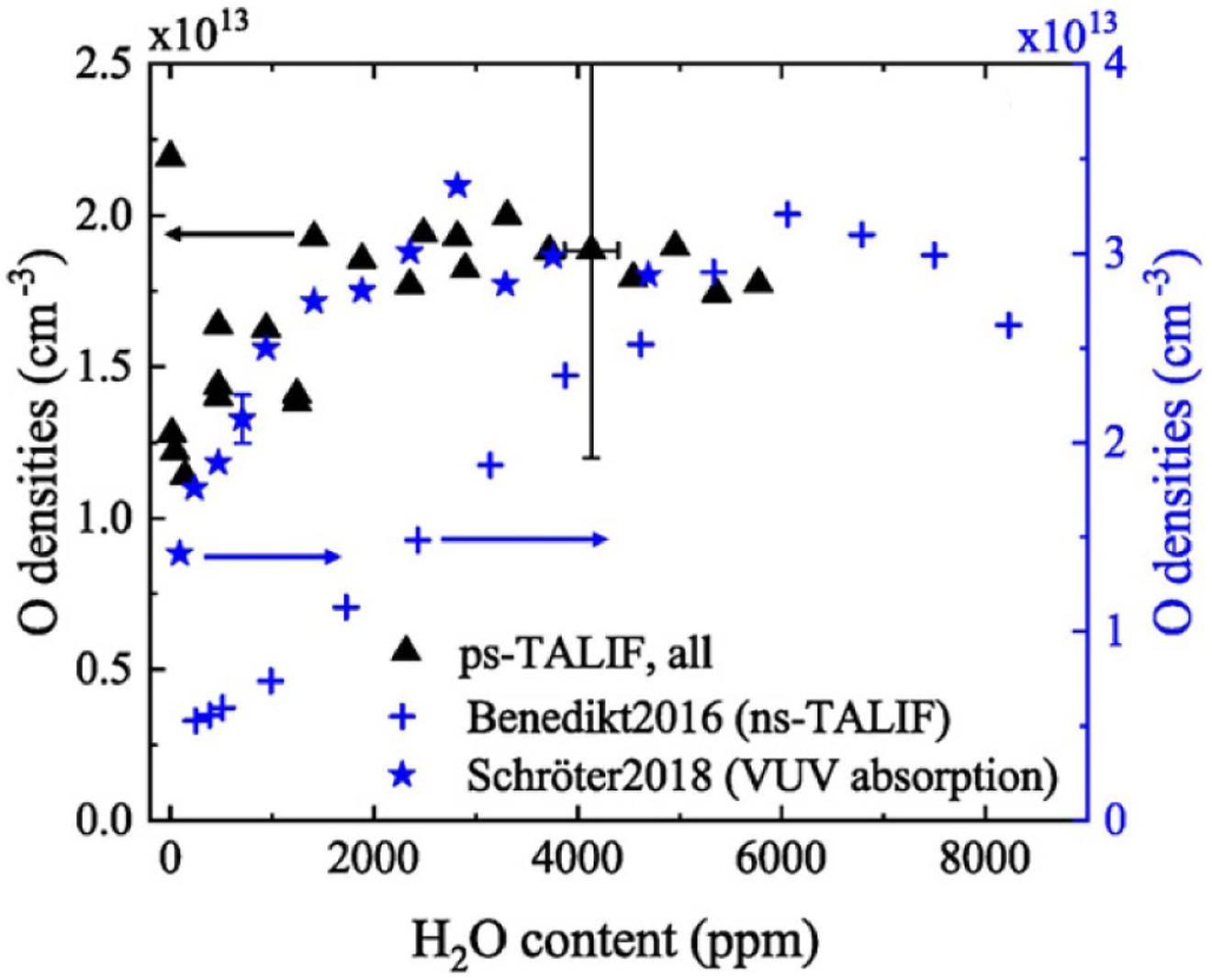
Comparison of measured absolute atomic oxygen densities in similar plasma sources using different diagnostic techniques: ns-TALIF [36], synchrotron VUV absorption [10] and ps-TALIF [37]. The plasma sources operate in a helium carrier gas flow through a 1 mm discharge gap with varying humidity admixtures. The power delivery is a RF-CCP at 13.56 MHz. Reproduced from [37]. © The Author(s). Published by IOP Publishing Ltd. CC BY 4.0.

All three techniques used in this comparison have different advantages and disadvantages with associated limitations and uncertainties. These differences are important to consider in assessing the results of the benchmark study in addition to the slightly different plasma sources and potential differences in the measurements introduced by those differences in sources. Measured absolute atomic oxygen densities for varying humidity admixtures obtained using ns-TALIF [36], synchrotron VUV absorption [10] and ps-TALIF [37] are shown in figure 3. In general, there is good agreement across the three techniques with there being best agreement between VUV absorption and ps-TALIF. The differences compared to ns-TALIF are systematic and are likely explained by the needed estimate of collisional de-excitation rates based on quenching coefficients [37]. The ns-TALIF and ps-TALIF measurements were calibrated using the same method using xenon as reference gas [39]. Given this calibration, in principle, the difference between the two TALIF techniques lies with the analysis. Recent studies and direct measurements of the xenon two-photon absorption cross section suggest that there is an additional systematic uncertainty in this calibration technique [40, 41]. However, this uncertainty only affects the absolute densities and should not account for the relative differences. That said, there is also a discrepancy between VUV absorption and ps-TALIF at low humidity admixtures. Details of the discrepancy are discussed below.

**Figure 4.**
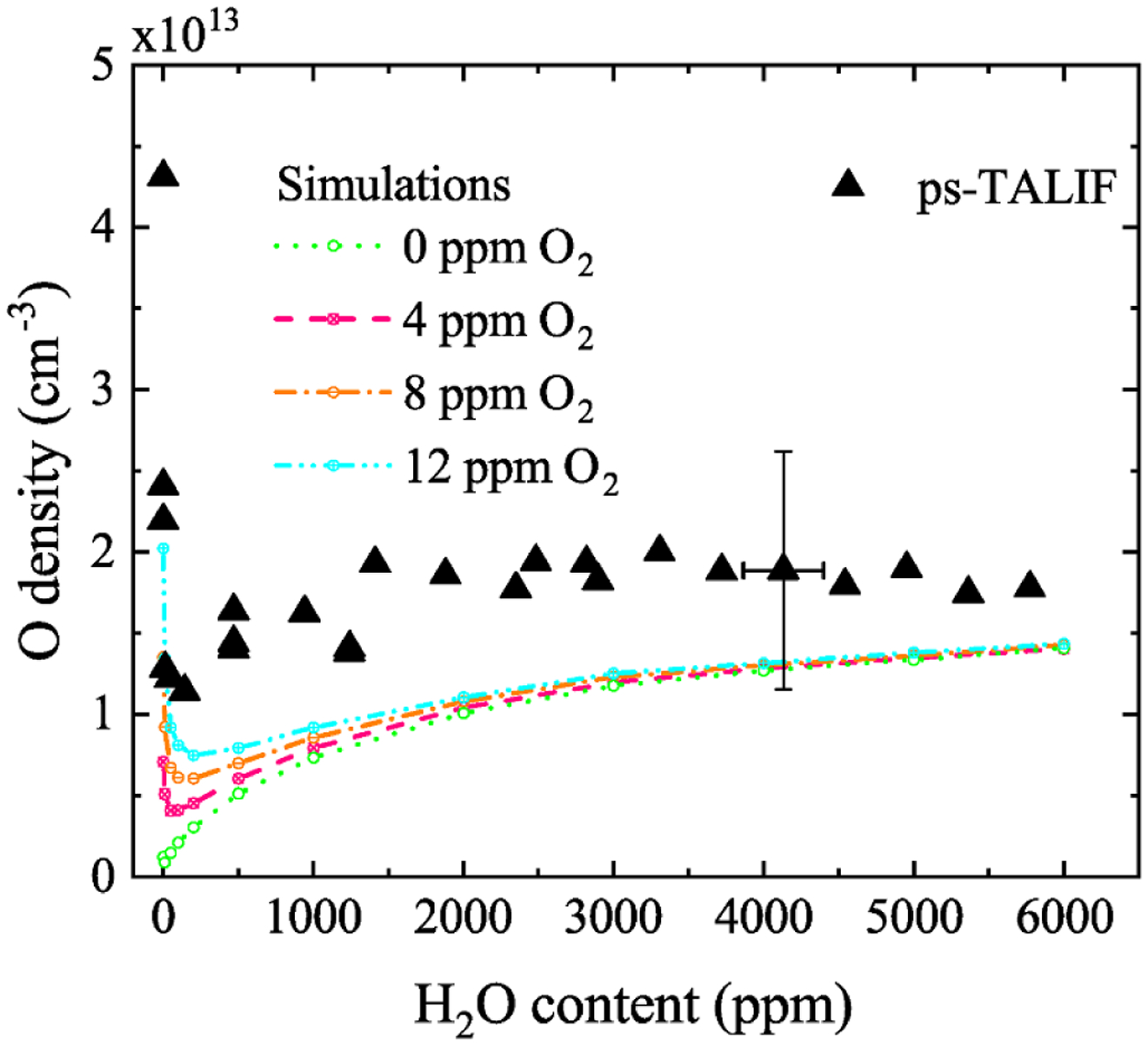
Comparison of experimental measurements of absolute atomic oxygen densities and computational simulations as a function of humidity admixture. Experimental measurements were carried out using ps-TALIF. Computational simulations were based on GlobalKin [43] using different levels of O_2_ impurities. Reproduced from [37]. © The Author(s). Published by IOP Publishing Ltd. CC BY 4.0.

A comparison of the ps-TALIF measurements with computational simulations as a function of humidity admixture is shown in figure 4. The computational simulations were based on global model assumptions [42] using different levels of O_2_ impurities in GlobalKin [43]. Details of the simulations and the reaction mechanism are in [37]. There is generally good quantitative agreement between simulation and experiment within experimental uncertainties as indicated by error bars in the figure. Without oxygen impurities (0 ppm O_2_) the simulation predicts a continuous increase of atomic oxygen as the humidity admixture increases. This behavior agrees with VUV absorption measurements carried out under strict vacuum conditions [10] as well as with ns-TALIF measurements carried out in a controlled helium atmosphere [36]. Particularly interesting is the influence of small O_2_ impurities (4–12 ppm) at low humidity content. In this regime, the simulation predicts significantly elevated densities of atomic oxygen from dissociation of O_2_ impurities. These elevated atomic oxygen densities agree with ps-TALIF experiments operated at ambient laboratory conditions, typically influenced by small impurity levels [37].

This case study demonstrates that benchmark experiments for different diagnostic techniques may have an uncertainty envelope. Validation with computational simulations can provide synergy with additional mechanistic insight. This specific case study also demonstrates the potential importance of impurities. The development of standard protocols should include specified impurity levels (or dependence on impurities), as should also be the case in development of reference plasma sources, as mentioned in section 2.

### 3.3. Recommendations for standards in plasma diagnostics

In contrast to other disciplines, such as engineering, chemistry and biology, recommended protocols for standard measurement techniques are less developed in plasma science, and for LTPs in particular. This situation is largely due to the challenging and strongly diverse nature of LTPs, often requiring diagnostic techniques specifically adapted to the distinct plasma environment and particular plasma parameters of interest. On the one hand, this makes the development of standards for LTP diagnostics a complex endeavor. On the other hand, it is important to understand the limitations of different diagnostic techniques, associated measurement regimes and uncertainties. The development of standards can provide clear benefits in transparency, reliability and transferability of experimental measurements. At the same time, standards should minimize additional cost in terms of additional infrastructure and time investment. These standards should not result in unintended constraints and generate potential barriers for developing and deploying new diagnostics; or applying current diagnostics to new configurations and parameter spaces. To the contrary, the development of standards should be a dynamic ongoing process and support future developments.

Dedicated review or tutorial style articles can be a good starting point for proposing and developing reference standards. This process could begin fairly narrowly in scope, addressing particular diagnostic techniques used to measure selected plasma parameters in a plasma environment of interest. Valuable articles already exist in the literature in this regard, and should be surveyed as a basis for establishing standards. That said, review articles usually focus on previous research while there is a need for survey articles with a focus on recommendations for establishing standards and protocols for forward looking activities.

Key issues to consider in a proposed framework for diagnostic standards to support transparency, reliability and transferability of experimental measurements are:

- Identifying and classifying the diagnostic technique.
- The regime of validity of the technique (that may evolve over time).
- Analysis technique(s) or requirements for a range of plasma environments.
- The range of equipment and potential calibration procedures.
- Input data required for analysis of the data, including uncertainty assessment.
- The consequences of impurities.

The implementation of such diagnostic standards could be supported by cataloguing community-accepted hardware and software tools and guidelines for their use, as has already been realized with the COST-jet. Care should be taken in developing these catalogues and recommendations for equipment since the situation here is different than with plasma sources. The development of standard plasma sources has been, to date, a bottoms-up community effort producing a unique, and at least initially, non-commercial device. Most sophisticated diagnostics, and laser-based diagnostics, employ purchased commercial equipment. Standards should avoid endorsing a particular company’s products. Rather, the standards should recommend the equipment’s specifications (e.g. wavelength range, bandwidth, energy, pulse length, resolution). Doing so may motivate more companies to develop or offer products having these specifications.

The discussion of diagnostics in this section is intended to cover the entire diagnostic infrastructure. For example, it is not only the capabilities of the laser in a LIF diagnostic that requires specifications and description, but also the detectors, spectrometers and critical optics. The latter would be quite important in, for example, Thomson scattering where suppression of the Rayleigh scattered light is important.

In the absence of established standards, it is recommended that in their reporting, researchers follow a similar general framework as described here. Doing so supports transparency, reliability and transferability of their results. A clear and detailed description of the equipment used for the diagnostic, the precise implementation of the diagnostics (e.g. voltage sweep speed in a probe measurement, repetition rate for a laser), the input data to the analysis and the analysis techniques should be provided. One example of such a framework is the initial draft of the plasma metadata schema [44]. Although this discussion has emphasized processing of diagnostic data, a key element to transparency is to also provide access to the raw data, which would allow potential reanalyzing of data using different techniques or when analysis techniques are improved. Enabling access to raw data is already strongly encouraged or required by several international funding agencies and journals.

### 3.4. Links to other sections for plasma standards

There are close links between diagnostics standards with standards discussed in other sections in this article. Standard plasma sources (section 2) require reliable diagnostics for accurate characterization. In turn, well-characterized standard sources provide an ideal platform for the development of new diagnostic techniques as well as benchmarking of different diagnostic techniques against each other. Benchmarking is a particularly powerful tool when different techniques rely on different analyses techniques or assumptions and use different input data. Benchmarking can provide an uncertainty envelope and identify weaknesses in model assumptions and input data (sections 4 and 6). Standard plasma sources and accurate characterization using reliable diagnostics are also key in the development of potential concepts for developing a plasma dose (section 9). Data obtained from measurements should generally be made openly accessible, including raw data (section 8). Analysis techniques for diagnostic measurements should be transparent and ideally based on open source analysis codes (section 7).

## 4. Data and mechanisms

Over the past decade, the topic of input data for computational models and simulations of LTPs has taken on greater importance and experienced increased activity within the LTP community. The community has realized that reliable and validated data is critical to the use of models in both investigating processes in established parameter spaces and extending models into yet-to-be-experimentally investigated parameter spaces. This increased interest has resulted in community-driven projects such as LXCat [45, 46], Phys4Entry [47] and VAMDC [48, 49], which recently celebrated their tenth anniversaries, and in commercial offerings such as the Quantemol database QDB [50]. Another indication of the importance of the topic is the level of activity at dedicated conferences such as the International Conference on Atomic and Molecular Data and Their Applications (ICAM-DATA), International Conference on Data Driven Plasma Science (ICDDPS) [51] and the recurring topical sessions on plasma data in more broadly attended meetings such as the GEC [52]. The importance of input data has also been acknowledged by publishers. It is becoming more common that scientific papers provide a comprehensive listing of all input data that have been used for a particular investigation. One such example is in the form of digital auxiliary data files hosted on the publishers’ websites or in third-party repositories such as Zenodo [53-55].

Input data have always been crucial ingredients in plasma research. The recent change in that emphasis is that the focus is no longer solely on the data but also on formats for exchange of data, and reliable and convenient web-based dissemination protocols. Examples of projects whose goals are, in part, to provide such access to atomic and molecular data are VAMDC and the XSAMS document format [56] upon which it is based. Another example is INPTDAT [57], which has a broad scope and promotes the FAIR (findability, accessibility, interoperability, and reuse) [58] data principles for scientific communication. LXCat is another example for data dissemination [46]. LXCat has become a de facto standard for cross section data, in particular for electron-impact processes, and for standardizing input file format.

Is this realization of reliable communication channels the predecessor of a standardized workflow in LTP physics? It definitely is a prerequisite, however standardizing a workflow has other requirements as well. Adoption of a new workflow will occur only if there are demonstrable advantages for the producers and end-users of the data. Since many plasma regimes and types of sources fall under the LTP umbrella, each with particular data needs, it is not clear that a single standard will ever be able to meet the needs of the LTP community at large. Any such effort to establish LTP data standards also calls for an understanding of existing standards in adjacent fields, such as quantum chemistry, transport physics and chemical reactor engineering. This section touches on these issues, but starts with what is perhaps the most important question of all: ‘Why do we need standardization of data in the first place?’

### 4.1. The desirability of standards development

The first issue that needs to be discussed is what can be gained from the development of input data standards in LTP science. There are at least two aspects of data standards that should be considered:

- Standardization of data representation and methods for data handling.
- The establishment of standard (reference) data sets.

Some immediate advantages of the adoption of standard file formats will first be discussed from the perspective that the real challenge of standardization is in the clarification of terms and labels. The complexity of a simple question such as ‘what is the meaning of N_2_?’ will be discussed by contrasting the state-to-state approach to plasma modeling with the use of self-consistent electron data sets that is common in gas discharge physics. We will consider the pros and cons of establishing reference data sets.

When new plasma simulations are developed that use or develop a particular physical model of the plasma, extensive testing should be done to verify the code’s correctness. This is usually done by a combination of testing of individual subunits of functionality and comparing results of the full model with well-established previous results, obtained either as analytical (asymptotic) solutions, experiments or from numerical calculations. In the latter case, the task requires that the same input data and operational settings are used as in the reference study. In doing so, any differences in the results can be attributed to differences in the algorithms used in the reference work and in the new code. This type of benchmarking is good practice in computational sciences. Examples of benchmarking in other fields include a collection of test problems for matrix solvers (the Matrix Market [59]) and test problems for computational fluid dynamics (CFD) [60]. A developer of a new code will typically test the code against the reference cases. Although this testing is part of the culture of the CFD community, there are no requirements for such testing by the leading journals of the field.

In plasma science such rigorous benchmarking appears to be less of a standard practice, at least in the published literature, but there are notable examples of such community benchmarks. For example, five independently developed particle-in-cell (PIC) codes have been compared in a detailed benchmark study by Turner *et al* [61]. The Landmark project [62] provides a platform for comparing low-pressure magnetized plasma codes. In a more recent study [63], six codes for simulating positive streamers in air were compared. The level of detail in the specification of the cases varies in these examples. However, all followed the practice of specifying a relatively small amount of necessary input data, so that emphasis would be on the algorithms.

When testing a new code relies on large amounts of input data (e.g. species properties, radiative transitions, reaction mechanism), setting up the test case can become a laborious task, especially when the data have to be reconstructed from tables, graphs and informal descriptions in publications. Interpreting and collating these sources of data inevitably involves individual judgement in issues as simple as the number of data points that are extracted from a line graph, which then impacts the interpolation of that data during execution of the code. As an example, the LoKI-B code for solution of Boltzmann’s equation for electron energy distributions [64, 65] (see also section 7) has been rigorously bench-marked against the BOLSIG+ [66] code. In order to use the same data in both codes, additional information was required on the specific excited states corresponding to the electron impact excitation cross sections used in the original input files of BOLSIG+. Since the codes adopt slightly different models (e.g. to obtain an elastic cross section from effective momentum transfer cross sections) and different levels of detail in the number of excited states, the exercise of benchmarking was limited to simple cases. In another example, at the bi-annual ‘Non-LTE Code Workshop’ (see for example [67]), harvesting and interpreting the correct input data appears to be part of the challenge that is posed to the participants. Finally, an effort [53] to simulate a CO_2_ plasma with dozens of species and thousands of reactions with two different codes and comparing the results has been successful, but revealed the difficulty and intensive labor required to accurately reproduce results of previous works. This state of affairs goes against one of the key principles of science—reproducibility.

In other fields a portion of the challenge of reproducibility has been addressed by establishing (reference) data sets and distributing them in electronic form. An example from a nearby field of science is the GRI-Mech (Gas Research Institute) mechanism for the combustion of hydrocarbon gases [68]. The availability of the data set as a computer-parsable file not only addresses the problem of ambiguity, but also mitigates the risk of introducing typing or unit mistakes in the assembly of the test problem. In the absence of such well-established (and named) reference sets, strict testing of codes for plasmas in complex gas mixtures becomes difficult to the point that it is not frequently done, or that differences in output due to unexplained reasons are taken for granted [63].

In emphasizing the importance of reference data sets for benchmarking codes, we have not focused attention on whether the data is physically valid. If the interest is simply benchmarking codes, the best data for comparing results may in-fact be an artificial test suite. However, more interesting reference sets describe an actual physical problem for a particular parameter range, a good example being the GRI-Mech mechanism. Such a mechanism can be used as input by an end-user who addresses more applied issues, for example a new burner design. Such separation of concerns is becoming more relevant as we experience ‘democratization’ of computational plasma physics. Whereas in the past simulations were usually performed only by plasma-computational experts, today computations are widely performed by others, for example for engineering purposes, using commercial and non-commercial codes. This is a wonderful development—more computations are being performed by more researchers. However, with the computations being done by non-experts, there is also the risk of reduced rigor in the selection of mechanisms and data when the focus is elsewhere and if no reference data sets are *a priori* defined. On the other hand, the availability of ready-to-use mechanisms may hamper original research and debate about the relevance of species and reactions in the system at hand.

### 4.2. Exchange formats

Standardizing the digitally available files for the representation of data and the methods for data handling is an important aspect for the dissemination of plasma input data. Reading such files should not be a source of confusion and errors itself. Using a standard file format such as XML or JSON relieves the users from the need of programming custom lexers or parsers, with the usual difficulties in getting all of the details correct. Even the apparently simple task of reading a two-column data set from an ASCII file, for example one that represents a cross section as a function of energy, is non-trivial if one cares about the details. Among the difficulties are the handling of local settings (e.g. is a comma a thousands separator, or a decimal sign), being prepared for DOS and Unix line endings, the handling of missing or superfluous data on a line, the identification of different blocks of data and the precision with which the data is written and read (single or double precision). Such a file cannot be automatically processed if the units cannot be reliably inferred from the document. Depending on the task at hand, many additional meta-data may (or should) be required, such as a statement on the accuracy of the data, the method by which that data have been obtained or how extrapolation should be carried out to obtain values outside the range of the table. It is also essential that references to underlying literature can be easily extracted so that proper credit is given to the authors of these publications. As the complexity of the document increases (more fields, data in logical sections, perhaps in recursive format), the complexity of a parser increases. For a typical example we refer to the file Code/Parse.m of the open source LoKI-B code [64, 65], which contains the code for parsing an LXCat-style input file. In such cases the advantages of a standard structured file format become even more obvious.

XML has been adopted as the exchange format by the VAMDC consortium [48]. In the case of LXCat [45], XML output is available as an experimental feature, while a switch to JSON is being considered as part of a reimplementation of the software stack [46]. We are not aware of resistance against the adoption of structured document formats per se, but acknowledge common concerns. The format should be vendor-neutral, and software libraries must be available for the most common platforms. Both XML and JSON match these criteria, though they are not the only options. It is also important for acceptance of a new format that backward compatibility is ensured, by providing software that translates new-style documents into prior formats so they can be used with existing software.

### 4.3. Standardization of state-to-state data, XSAMS and VAMDC

The choice of a structured document format should not be confused with the choice of a *particular* format, such as XML or JSON. The importance of that latter choice is sometimes over-emphasized, since lossless conversion between files of such types can be accomplished with standard tools. It is more important that there be a formal, generally accepted specification of the contents of such a file, so the integrity of a document can be checked by a computer and a computer code can rely on particular data being available in the file. In the case of XML and JSON files, so-called ‘Schema’ can be used for this purpose [69, 70]. The real work of standardizing an input data document type reduces to the creation of the schema file for such documents.

The XML schema for atoms, molecules and solids (XSAMS) project [56] is an example of an (XML) schema for atomic and molecular data. The schema was introduced in 2009 and is supported by a consortium that involves organizations such as the International Atomic Energy Agency (IAEA) and the National Institute for Science and Technology (NIST) in the United States. XSAMS has been adopted as the primary output format by the VAMDC project, a community-driven e-science infrastructure that provides access to dozens of databases. The result is that such data can now be obtained via a uniform query interface and that the XML files that are produced have the predictable structure defined by the XSAMS schema. For a recent overview of the databases connected to the VAMDC infrastructure, refer to table 1 of [49]. The emphasis of VAMDC is on spectroscopic data which resulted from the involvement of astronomers, for example through the International Virtual Observatory Alliance (IVOA) and the Observatoire de Paris in the project.

Collisional data are also available in VAMDC, and among the many examples are the BASECOL2012 database [71]. The VAMDC project has a formal approach to define the states of atoms and molecules (see, for example [72]). The format is best-suited for processes involving states for which a complete set of quantum numbers is available (‘state-to-state data’), and the BASECOL2012 database is an excellent example of such a data set. Another example of a collection that contains state-to-state data is the Phys4Entry database [47]. While the number of identifiers that is needed to define a state is large, the advantage of this type of data is that there is little room for semantic confusion, which is common when adopting ambiguous designations such as Ar* when referring to an excited state of argon (see below).

The state-to-state approach has some additional advantages. Among others, the data items (such as radiative transitions and collisions) are elementary, and as a result also context-free. This means that, for example, data on collisions in a hydrogen plasma can be combined with data on a nitrogen plasma when a hydrogen–nitrogen mixture is simulated. Only the ‘cross-terms’ that involve both hydrogen and nitrogen species must then still be added. Data obtained from calculations are usually of this state-to-state type. Depending on the application, vast amounts of such data might be needed, for example for the detailed representation of an emission spectrum or for the calculation of the thermodynamic properties of a plasma in a molecular gas in local thermodynamic equilibrium (LTE), such as partition sums and specific heat. The detailed information on the states and the systematic representation of the data in a standardized form such as that offered by VAMDC then facilitates the implementation of such tasks in a computer code. Examples of codes that can operate with VAMDC are SPECVIEW [73] and CASSIS [74], both spectroscopic tools. For more software using the VAMDC schema, see the list in [75]. However, the level of detail enabled by the state-to-state description and the subsequent effort in handling large amounts of such data might be excessive when simpler models suffice. Standard input data formats, referring to different reference data sets, should be able to accommodate the different needs of the users.

### 4.4. Mechanisms

Reality is often not as elegant as the state-to-state approach suggests. In LTP physics, cross sections may have been obtained from drift tube experiments and may describe more than one elementary process. For example, the excited states of a species may be lumped into a single species such as Ar* and an atomic argon plasma is then represented as mixture with species Ar, Ar*, Ar^+^ and electrons, for example. [Lumped species, or multiple lumped species (Ar*, Ar**, Ar***) may be necessary to reduce the complexity of a simulation to a manageable level.] The energy levels and the excitation cross sections in such a model may be the outcome of fitting experimental data within a theoretical framework such as a two-term spherical harmonic solution of Boltzmann’s equation for the electron energy distribution [64-66]. The cross sections are tuned such that experimental values of drift and diffusion coefficients and inelastic rate coefficients are accurately reproduced by the model. Such cross section data are not elementary in the sense that they do not describe individual transitions. As a consequence, such swarm-derived data sets should be combined with other data with caution. That said, momentum transfer cross sections and transport coefficients such as for mobility and diffusivity for many species are only available by unfolding swarm data in this manner. For more details refer to section 5.4 of [46].

By using lumped-states or effective-states such as Ar*, the amount of data that is needed by a model in order to get accurate results for a plasma is smaller than a complete state-to-state model. The cost is semantic confusion. For example, what is the meaning of Ar* or N_2_? Does a species with the name N_2_ include all the states of this molecule or only the rovibrational states of the electronic ground state? The specific intent of a species named Ar* or N_2_ should be made clear in the publication of results. However, the same species Ar* or N_2_ could be defined differently in different papers. Removing such ambiguity could be accomplished by a community accepted definition of Ar* or N_2_ and capturing the definitions in a standard. Ideally, a lumped state such as Ar* would be defined as the combination of specific, spectroscopically defined states, along with the method of combining those states and their electron impact cross sections. This is particularly important for super-elastic and quenching coefficients. The current practice is to typically add the electron impact cross sections of the individual states for excitation of the lumped state, but use a single cross section for quenching since there is only the single state. This practice has not rigorously been tested nor standardized. In particular, what is the degeneracy for a lumped state, an important consideration in computing super-elastic cross sections? In the LXCat community experiments are going on with a data model that accommodates these types of lumped state data [46]. The VAMDC community appears to acknowledge this issue (see the discussion on ‘fuzzy matches’ in section 3.3.2 of [49]).

The complete data sets for electron-impact cross sections that are offered by LXCat, called *xs-sets* for brevity here, follow an ontology similar to the reaction mechanisms that appear in other fields, like GRI-Mech in combustion science. Reaction mechanisms are collections of data (reactions, rate coefficients, thermodynamic quantities) that produce good results for a reasonable computational price. Reaction mechanisms are not universally applicable and so the parameter spaces for which they are valid should be specified as part of the mechanism. For example, in combustion this usually means a range of gas temperatures, pressures and initial gas compositions. In the case of the *xs-sets* offered by LXCat, the cross sections alone are valid for describing the electron kinetics of gases or mixtures of gases where there is negligible interactions between the gases. When there are interactions between gases, the *xs-sets* can be included as part of more complex plasma models with a full reaction mechanism to address applications at higher gas pressures and temperatures, and under non-LTE conditions. Only a few of such reaction schemes are available publicly in electronic form, and then often only in custom file formats [53, 55]. Many mechanisms that are of immediate use are also offered commercially by QDB [50], also in a non-standard format such as qdat, and formats that enable use by a small selection of non-commercial codes, such as hybrid plasma equipment model (HPEM) [76].

### 4.5. Conclusions

We have discussed the motivation and possible process for developing a standard for plasma input data, and discussed that the data needs of the astronomical, high energy density, LTE and LTP groups of the plasma physics community are quite different. A common standard seems worth the effort, but requires a data model that accommodates state-to-state plasma data as well as mechanisms. Such a standard should not ignore the existing tools and data-formats, particularly in cases where they have wide acceptance. The adoption of a standard or standards will strongly depend on the advantages that are experienced by users. Such advantages can come in the form of applications or postprocessing facilities that operate on the data, such as the spectral software that is available for VAMDC or Boltzmann solvers that can work with LXCat data. Simplified search and re-use of relevant data (e.g. for validation of models, comparison of results, or conducting meta studies) may lead to broader acceptance of the FAIR data principles and sharing of digital data sets with comprehensive and standardized data descriptions, one example being that intended by the data platform INPTDAT. This would open up new possibilities for linking data to associated plasma sources, applied diagnostics and software packages used. In this discussion, it is important to separate the technical aspects such as using standard file formats from the discussion on the content of standard and reference data sets.

## 5. (Quick) Data generation

Models of LTPs rely on knowledge of the various chemical processes that can occur in the plasma. Such processes are broadly of two types: the interaction of electrons with atoms and molecules, and heavy particle collisions which give rise to chemical reactions and related processes. Depending on the plasma chamber, processes which occur on surfaces may also be an important driver of the overall composition of the bulk plasma. Indeed, these surface processes are often the reason for studying the plasma in the first place. As constructing a full chemical model for a plasma can require a significant amount of data for a range of different processes, theoretical methods are increasingly the means for providing such data [77]. Data can be sourced from databases (see section 4) but often it is necessary to complete datasets by generating extra data using quick but approximate procedures.

These quick methods should also enter into the discussion of standards and best practice. Most complex plasma chemistry models, by necessity, make approximations, analogies and estimates to fill in values for processes for which there are no experimental or computed data for cross sections, mobilities and transition probabilities. In reporting on these models, the rationale for making these approximations should be discussed. That said, a community consensus on recommended practices to fill in the missing data would enhance our ability to assess the results of the models, to understand the rationale for making these approximations and to lend systematic consistency between models. This section sketches some of the procedures to quickly produce these data, available starting from the simplest and moving to more sophisticated and computationally demanding methods.

### 5.1. Scaling laws

Perhaps the simplest means of generating new data quickly is the use of scaling laws. Scaling laws are widely used in plasma physics and in the context of constructing chemical networks, scaling laws can be performed based on a number of different properties. For example, one can use the mass of the species as a scale factor which has a particular use for fusion plasmas. Data are often available for processes involving H and sometimes for D, but are rarely available for T because of the extreme difficulties of performing experiments with this radioactive species. Scaling laws for this situation have recently been proposed by Belli *et al* [78]. Similarly scaling laws for vibrationally-resolved molecular processes have been developed based on vibrational quantum numbers [79]. Other possible parameters that one can scale on are ionization stages for heavily ionized atoms and reaction classes where species come from the same group in the periodic table. Looking to the future, in many cases it should be possible to use the techniques of machine learning (ML) to generate new reaction rates using the ideas behind scaling laws but allowing for greater freedom in the parameters choices. This provides the possibility that given an appropriate training set, ML would provide a proper range of parameters to generate estimates for reactions whose rates are not known.

### 5.2. Electron-driven processes

There are simple or relatively simple formulae that provide rates for a number of physical processes that are important in plasmas. Perhaps the simplest of these are for electron impact rotational excitation of molecules which contain permanent dipoles. In this case there are standard analytic formulae for the cases of both neutral [80] and ionized molecules [81]. These formulae use the Born approximation which assumes that the interactions are all essentially long range. They are found to work well for molecules for which the permanent dipole moment, which dominates long-range interactions, is large with, for example, a value of 2 Debye or more. A similar formula is available for dipole-driven electron impact vibrational excitation of molecules [82]. However, this form is only appropriate for cases where the incoming electron does not get trapped in a long-lived, quasibound resonance state. As such resonances can increase vibrational excitation rates by many orders of magnitude the Born approximation formula for vibrational excitation should be applied with care. In fact, resonances also provide the route to dissociative electron attachment (DEA) which is a key process for the formation of negative ions in molecular plasmas. A procedure to estimate DEA rates is available [83] although this requires estimates of resonance parameters as part of the input.

The Born approximation can be used to estimate electron impact electronic excitation cross sections for so-called optically allowed transitions which are the ones driven by dipoles and are the only excitation cross sections which are important at high impact energies. The so-called BE*f* method involves use of a scaled version plane-wave Born approximate where the *f* in the acronym represents the oscillator strength of the corresponding optically allowed transition [84]. The BE*f* method becomes increasingly accurate for higher energy collisions where other, short-range interactions become increasingly less important and electronic excitation processes which do not depend on dipoles are not important.

Another higher energy process, electron impact ionization, has been the subject of study by a variety of different approximate and quick to apply methods. The most widely used of these is the binary encounter Born (BEB) method of Kim and Rudd [85] which has been found to perform reliably for a whole range of species [86]. Work is now currently focusing on how BEB, or indeed any of the other related methods, can be extended to predict fragmentation patterns for the resulting ionized species [87]. In a similar vein the Drawin approximation [88, 89] is used to give electronic excitation (bound-bound) and ionization (bound-free) rates for electron collisions with atoms although this method often gives an overestimate requiring the use of appropriate scaling factors to give useful results.

### 5.3. Heavy particle collisions

It is common to represent the rate coefficients for reactive collisions in Arrhenius form. This form is designed to capture the physical behavior of most chemical reactions in that they have a barrier to reaction that must be overcome in order for the reaction to proceed. However, there are classes of reactions, particularly ones involving the reactions of ions with neutrals, for which there is no barrier to reaction. The formation of H_3_^+^ in partially ionized hydrogen gas by the process H_2_ + H_2_^+^ → H_3_^+^ + H is a good example of such a process. These reactions often proceed with every collision which corresponds to the Langevin rate of the process, something that can be easily calculated for a given set of conditions. The Langevin rate therefore provides a good starting approximation for these fast processes. In the case of the reactions with a barrier, transition state theory [90] provides a relatively simple method obtaining reaction rates without need to study the dynamics of the atoms involved in the reaction.

## 6. Verification and validation (V&V)

Computer models of LTPs must meet at least two requirements to faithfully represent an experiment or investigate an experimentally unexplored region. The first is the selection of the proper formulation of the physics, which in LTPs would also include the proper reaction mechanism. For this discussion, we will call the formulation of the physics and the reaction mechanism the *equations*. The equations are transformed into computer code using mathematical techniques such as discretization on numerical meshes and integrated using, for example, linear algebra routines. The process of ‘validation’ is determining whether we have chosen the proper equations (including the reaction mechanism) to represent the physical phenomena. The process of ‘verification’ is determining whether we have solved the equations properly. No method in general use can prove that a computer program is free from error. The challenge, therefore, is to demonstrate that the results of a scientific enquiry are valid in spite of this limitation. The formal process of removing errors is a large part of verification.

Investigations of source codes for various technical applications have shown a variable level of success in the pursuit of verification, ranging from as many as one fault every ten lines to as few as one in every 10 000 lines [91]. From these observations, we might conclude that careful testing a code can reduce the frequency of errors by about a factor of a thousand. This reduction in the frequency of errors usually comes at a high price. The difference in the development time (and hence cost) between these extremes may exceed a factor of 10. So careful verification is expensive.

In recent practice, different fields have approached the verification challenge with different emphases. Engineering practice leans to a formal approach, with strong methodological prescriptions often being imposed, for example, by the editorial policy of journals [92]. Scientific communities have favored a less structured approach, which is sometimes characterized as allowing a major role for ‘expert judgement’ as a correctness criterion for computer simulations [92]. Suggestions are sometimes made that this approach reflects a lax attitude to verification on the part of some scientific communities. However, the aims of scientific and engineering calculations can be very different, and we should not ignore these differences. Allocating the very large resources required for serious verification may not make sense unless the benefit is clear. So why might scientists find the benefits of verification unclear?

In scientific work, a computer simulation is often a tool for exploring the qualitative behavior of a physical system. The aim of the work is not the development of the computer simulation, but rather the articulation of a higher-level understanding, often expressed as an analytical theory. A classic expression of this mode of work is found in the Fermi–Pasta–Ulam–Tsinghou problem, which began as a computer investigation of a model nonlinear system, but motivated a vastly fruitful field of enquiry into the general behavior of nonlinear systems, including the evolution of unprecedented mathematical tools [93]. In this and similar scenarios, the eventual outcomes are hardly dependent on the detailed correctness of the original computer simulations, which needed only qualitative validity. Further investment in verification would have added little value. A counter argument to this view is made by the requirements of scientific reproducibility. That is, there is the expectation that the procedure used in a scientific study is sufficiently documented that other researchers can reproduce the results. There is a risk that an insufficiently verified code does not actually do what the authors claim that it does. If this is the case, the results are irreproducible with there also being considerable confusion (and an associated waste of resources). This objection is much mitigated if the source code and other relevant data are published, but this is not often the case.

Very different situations arise in engineering [92]. Frequently, the aim of an engineering calculation is to calculate a number (the drag coefficient of a body, for example). Expert judgement cannot determine whether the number is correct or not, but crucial (and costly) decisions will be dependent on the number. In this context, formal verification and validation is the only available defense against incorrect decisions based on faulty numbers. The LTP physics community encompasses rather mature sub-fields, where the engineering approach to verification is likely appropriate, and exploratory areas, where a less formal approach may often be more efficient, so general prescriptions are difficult to find or define.

These extreme examples show that heavy investment in verification and validation is not always the best use of scarce resources. However, they also highlight the differences between these scenarios. A code developed for scientific exploration with little attention paid to verification is unlikely to be useful for engineering prediction, and should be used with caution in that context. The traditional practices of the scientific community for computations to provide qualitative predictions may not be adequate when the goal is to provide a consequential value or design. In this case, to some degree, the formal machinery of verification, validation, and uncertainty quantification becomes essential.

The responsibility of scientific investigators is to understand the role of computer simulations in their chain of argument. In this regard, an important question to ask is by how much could the results of the computer simulations be different without invalidating the conclusions? How much assurance is there that the results of the simulations meet this criterion? These considerations will often lead to the conclusion that some attention to questions of verification and validation is warranted. In that case, what techniques are available?

Verification and validation are distinct concepts [92]. Verification is concerned with the correctness of a computer code, that is, with demonstrating that the code works as intended. Validation is concerned with the correctness of the physical models. Demonstrating validity requires some form of comparison with experiments, but verification does not. Clearly, then, verification must come first.

**Figure 5.**
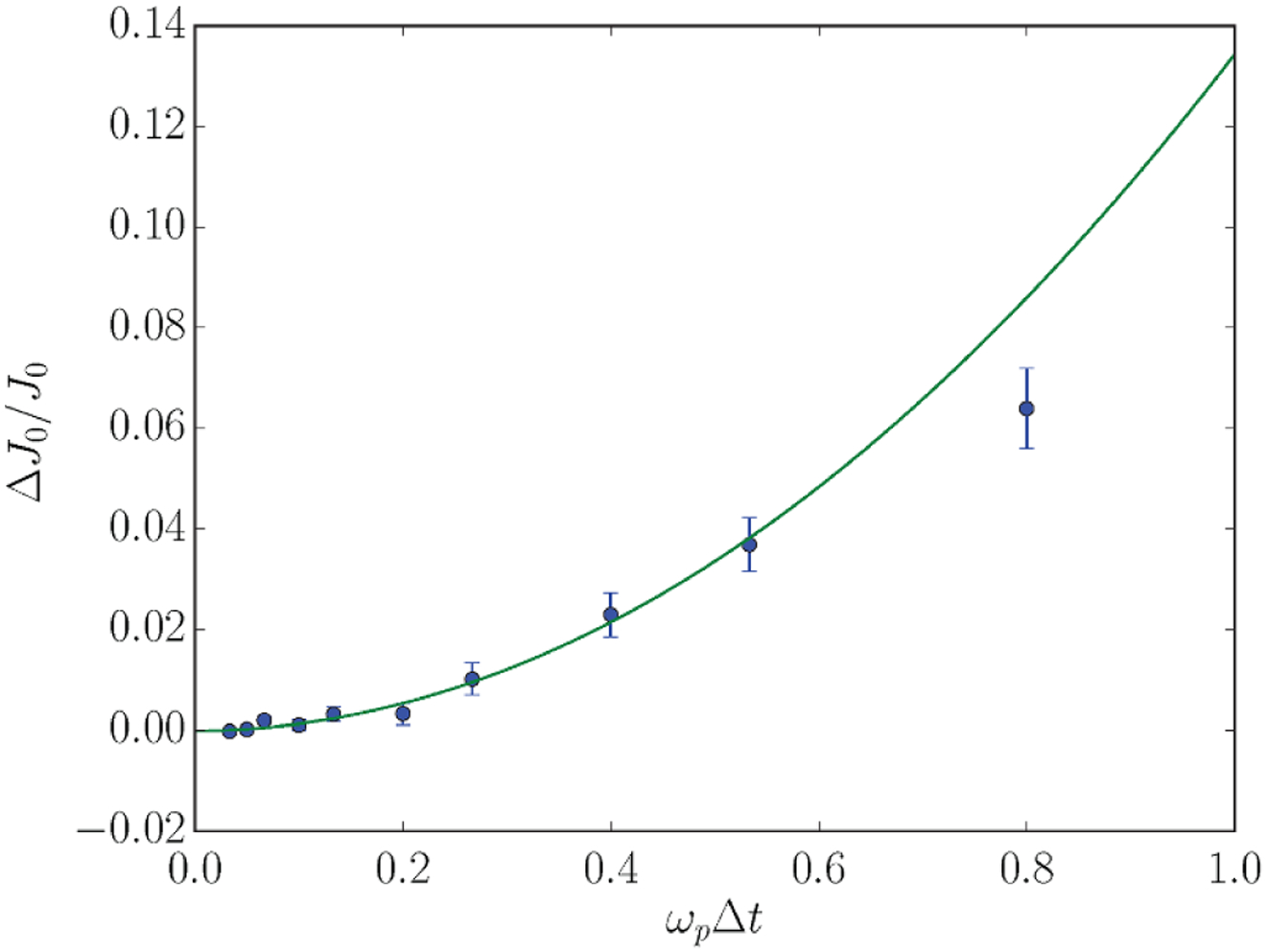
Verification by convergence toward an exact solution as a numerical parameter is changed. In this example, the physical parameter is the conduction current density flowing in a thermionic diode, and the numerical parameter is the time step in a particle-in-cell simulation. The points with error bars are simulation data, and the curve indicates the expected rate of convergence, which is O(Δ*t*^2^) in this case. Reproduced from [94]. © IOP Publishing Ltd. All rights reserved.

The canonical method of verification is to show that the computer code can reproduce an exact solution of the underlying mathematical model [92]. This comparison is not straightforward when both the ‘exact’ solution and the computed solution are calculated with finite precision. One method of quantifying verification is to measure the change in the distance between the computed solution and the exact solution when some numerical parameter is varied, such as mesh spacing or order of integration. This approach assumes that we can express the numerical error in the computed solution as a polynomial in the parameter, so we have an expectation as to how this distance should vary. This expectation will be realized only if the computed solution converges toward the exact solution at the expected rate. An instance of this technique is shown in figure 5. An objection to this procedure is that in any case where we know an exact solution, we might not be interested in verifying a numerical method. However, the method of manufactured solutions shows how an essentially unphysical solution can be constructed for verification purposes [92]. The premise of the method of manufactured solutions is that verification test solutions need not have direct physical relevance.

A corollary of this insight is that useful exact solutions may be found in unexpected places, for example in literature apparently remote from LTP science [94]. For some communities, such as CFD, this combination of techniques appears completely satisfactory. For LTP science, however, challenges remain, in that there are important classes of simulation where these techniques are difficult to apply. For instance, Monte Carlo methods (such as PIC simulations) often mix statistical effects with other numerical phenomena in a complex fashion, and are for other technical reasons resistant to the method of manufactured solutions [92]. Hybrid models typically use a non-standard mathematical structure, and may contain modules that use different algorithms [95]. Consequently, writing down a single coherent mathematical model for the purposes of verification of the entire code is difficult. For these and other reasons, the canonical methods of verification developed by the CFD community do not solve all the problems faced by LTP scientists. For example, establishing clear mathematical foundations for hybrid models does not appear at all easy, yet it is a prerequisite for formal verification. This does not mean that no progress can be made. For example, one can apply verification tools to individual modules of a hybrid model. However, a general verification framework for the central computational tools of LTP science is not yet available, and appears to present highly non-trivial challenges [94, 96].

An alternative procedure when formal verifications methods cannot be applied is benchmarking, which is a comparison of different computer codes applied to the same problem [61, 63, 92, 97, 98]. The difficulty with this procedure is that when the results do not agree (which is practically always the situation) there is no way of determining which solution is ‘correct’ if indeed any of them are correct [92]. This problem is again especially acute in the case of hybrid models, where there is not an agreed upon mathematical structure, and there is considerable room for variation in, for example, the choice of boundary conditions and the method of computing transport coefficients and injecting them into the moment equations. Consequently, differences between hybrid codes applied to a benchmark problem will usually arise from a mixture of error and the effects of legitimate alternative choices. A benchmark comparison cannot by itself disentangle these effects. The characteristics of the benchmark problem may also be important. For example, when the benchmark conditions exhibit physical instability, the computed solutions may depend appreciably on factors that are hard to control, such as implementation details like random number generators and aspects of parallel execution environments [98]. One might argue that a benchmark problem with such features is unsuitable, but a natural attraction toward conditions with obvious practical relevance weakens this objection.

Verification provides evidence that a computer simulation is correct, in the sense that the computer program delivers valid solutions to the underlying mathematical model, with a degree of uncertainty that can be characterized by investigating the influence of relevant numerical parameters. This is important, but does not introduce any evidence that the mathematical model appropriately represents any particular experimental situation. This can be demonstrated only by comparison of model calculations with experiments [92]. This process is known as ‘validation’. Excellent examples of this procedure are found in that part of the LTP science community concerned with the measurement and calculation of transport coefficients [99]. In careful comparisons, error bars are associated with both the experimental and computed data, where the errors in the computations arise both from numerical effects and from the presence of parameters with uncertain values. Uncertain parameters are treated by methods of ‘uncertainty quantification.’ The simplest procedure is to employ a Monte Carlo method to vary the uncertain parameters according to a suitable distribution [100]. This is a simple and direct approach, but certainly not the only one conceivable. But uncertainty quantification is a well-understood procedure. Clearly, the desired outcome of a validation exercise is that the measurements and calculations agree within the combined error bars. When such agreement is not found, identifying the cause of the failure may not be straightforward because there are so many possibilities. These causes are not limited to erroneous calculations: error (or misinterpretation) of the experiments needs to be considered as well.

These considerations show that a ‘one size fits all’ approach to verification and validation is undesirable and probably not possible. At one extreme, namely exploratory work intended only to motivate future investigations, detailed verification and validation are likely an inappropriate use of scarce resources. At the other extreme, engineering prediction often absolutely requires close attention to detailed questions of verification and validation. Of course, there is a continuum of intermediate situations. Always, the overriding principle is fitness for purpose. Investigators (and referees) should be clear about the aims of calculations, and careful to ensure that those aims are delivered by suitable attention to questions about ‘V&V.’

An important nuance here is the status of computer codes that are shared, by open sourcing or otherwise. In this case, the code authors do not always know how the code will be used. There have been instances where codes were employed (and improper results published) by code-users for parameter spaces that the developers of the code never intended. This notgood outcome may result from the algorithms not extending into that parameter space (verification) or the reaction mechanism not being proper in that parameter space (validation). The providers of codes should make it very clear the parameter spaces in which their codes and reaction mechanisms can be reliably applied, and users should take heed of those assessments. There seems in this case a responsibility on both developers and users of code to be alert to issues of verification and validation, but also an opportunity to share the burden.

There is a difficult path to navigate for both individual investigators and community actors such as editorial boards, in avoiding the extremes of imposing unreasonably and perhaps impossibly stringent criteria for V&V documentation, without causing or permitting confusion produced by poorly executed computations. There is wide scope for both technical progress and better understanding of the broader issues.

## 7. Open source and publicly available codes

Software development and computational calculations are prominent research activities in LTP science, yet surprisingly the community has not been driven to define clear standards for the various steps of the workflow, including the publication of results. The term *results* refers to both the outcome of the computational calculations and the code used in these calculations, since the code is necessary to reproduce, validate and confirm (or challenge) the calculations and the research findings [101]. Indeed, accessing the research software, as publicly available or open-source code, is not only desirable to ensure the quality standards of the published material, but it can also accelerate and inspire advances in the scientific work [102], especially in a small community such as LTPs.

The term ‘open-source code’ means any computer software under a license in which the copyright holder grants users the rights to use, analyze, modify and distribute the source code to anyone and for any purpose [103]. The term ‘publicly available code’ is used here in relation to any software that contains or is derived (in whole or in part) from ‘free software’, ‘open-source software’, ‘copyleft’ or similar licensing and distribution models; and/or when referring to any software redistributable at no charge for the purpose of making derivative works. Open-source codes are therefore publicly available codes, and this section addresses both, yet clearly distinguishing open-source codes as a special category of publicly available codes. We will start by briefly presenting some well-known open-source and publicly available codes in LTPs (including data generating codes), then making a case for open-source codes and finalizing with some recommendations for code development.

### 7.1. Examples of open-source and publicly available codes in LTPs

Although the LTP community has never defined a systematic route for the development of open-source codes or the sharing of simulation tools, this practice has been adopted by several members and groups for several decades. Some examples are listed below.

The problem of calculating the electron energy distribution function by solving the electron Boltzmann equation is among the subjects that most stimulated the development and sharing of codes. ELENDIF [104] (presently not available), BOLSIG+ [66], EEDF [105], BOLOS [106], and LoKI-B [64, 65] adopt the classical two-term approximation for solving Boltzmann’s equation [107-109]. METHES [110] and LoKI-MC [111] are Monte Carlo collision codes. Magboltz [112] uses a multi-term expansion (to the third order) of the electron distribution function with a Monte Carlo integration technique, and MultiBolt [113, 114] is a multi-term Boltzmann equation solver. Magboltz, BOLOS, METHES, MultiBolt and LoKI-B/LoKI-MC are open-source. Magboltz is a Fortran code with hardcoded data. BOLOS is a Python library using an algorithm similar to that adopted in BOLSIG+. METHES, MultiBolt and LoKI-B are written in MATLAB. LoKI-MC is written in C++. BOLSIG+, BOLOS, LoKI-B/LoKI-MC, METHES and MultiBolt accept input files with electron scattering cross sections obtained from the LXCat open-access website [115].

There are two very popular freeware codes to solve global 0D plasma chemistry models. GlobalKIN [43, 116], available upon request, solves a multi-zone model for plasma kinetics and plasma-liquid-surface chemistry, with electron rate coefficients calculated from a two-term spherical harmonics expansion of the electron Boltzmann equation, at *E/N* values provided by a circuit model or a power waveform. ZDPlasKin [117] is a computational utility for complex plasma chemistry that adopts a two-step operation. First, a preprocessor is used to translate the list of species, reactions, and rate coefficients from a user-friendly text format into a Fortran 90 module that interfaces to an ODE solver and the BOLSIG+ Boltzmann solver. Second, the compiled code calculates the time evolution of the densities of species and the reaction rates.

Often, plasma chemistry schemes involve several hundred (thousand) reactions, with very different impacts on the model results. The LTP community has started to adopt sensitivity analysis approaches to reduce these schemes, aiming for an easier definition of ‘reaction mechanisms’, corresponding to sets of reactions and rate coefficients validated against benchmark experiments. The open-source C++ code Pump-Kin (pathway reduction method for plasma kinetic models) is a tool for the post-processing of results from 0D plasma kinetics solvers [118]. The tool, compatible with the output format of GlobalKIN and ZDPlasKin, was developed to reduce complex plasma chemistry schemes, and can also analyze the production and/or destruction mechanisms of certain species of interest.

In a different approach, GlobalKIN was extended using the expert system Quantemol-P [119], for the automatic generation of the plasma chemistry which considers all possible gas phase reactions and the likely surface reactions, from a set of atomic and molecular species specified by the user. The reactions are sorted by importance, and the chemistry set is pruned by discarding unphysical reactions and reaction data. The system has been further extended, by adding the ability to generate electron-molecule collision data using Quantemol-N [120]. Quantemol-N is designed to treat low energy electron impacts, specifically those processes which lie below the ionization threshold of the species concerned, by running fully *ab initio* molecular R-matrix codes [121].

In the 90s, the freeware tool SIGLO-2D, a 2D user-friendly model for glow discharge simulation [122], was often adopted in the numerical fluid modelling of radio-frequency discharges. The tool, which is no longer available, was validated using measurements of the spatial distribution of the plasma density in the GECRC [123, 124], as discussed in section 2.

More recently, the HPEM [95], available on request, is a comprehensive modelling platform developed for low pressure (<a few tens of Torr) plasma processing reactors. The HPEM with a primary version in 2D and less supported version in 3D adopts the hierarchical approach of a hybrid modelling, in which different physical processes on vastly disparate timescales are addressed in compartmentalized modules (e.g. electromagnetics, electron energy transport, fluid kinetics-Poisson, plasma chemistry, surface kinetics, radiation transport), iteratively combined using time-slicing techniques. The HPEM has been applied to a variety of reactor types, for example ICPs, reactive ion etchers, electron cyclotron resonance sources, magnetron sputter and ionized metal physical vapor deposition, remote plasma-activated chemical vapor deposition, and dust particle transport. The HPEM is widely used in the semiconductor industry for plasma equipment and process design.

The same group that provided HPEM has also developed, validated and released the modelling platform nonPDPSIM, originally written to simulate plasma display panel (PDP) cells, but whose application space has grown in scope [125]. NonPDPSIM is a 2D multi-fluid hydrodynamics simulator in which transport equations for all charged and neutral species and Poisson’s equation are integrated as a function of time on an unstructured mesh capable of capturing a large dynamic range in length scale.

Recently, the Idaho National Laboratory (INL) has built the open-source software development framework MOOSE (multiphysics object oriented simulation environment) [126]. MOOSE is designed to solve highly non-linear, coupled systems of equations across various areas of physics and containing multiple physical models. MOOSE adopts a rigorous and well-documented development strategy, with a comprehensive set of tools for testing, so that changes to the code or MOOSE-based applications are only merged into the framework when the testing ensures that the changes are compatible with the applications. With this modular structure, users can either use the existing applications or develop new applications based on their needs. The LTP community has used this framework to model atmospheric pressure plasma-liquid interactions [127, 128], using the Zapdos-Chemical ReAction NEtwork (CRANE) open-source package that compiles the CRANE module [129] into the plasma transport software Zapdos [130]. An electromagnetic module, implementing the full set of Maxwell’s equations in the MOOSE framework, is currently being developed [131].

Another recent addition to the LTP simulation community is SOMAFOAM [6], a fluid-based LTP software platform built on the popular open-source CFD software OpenFOAM [132]. Like MOOSE, it is also modular, but built on a finite volume solver as opposed to finite elements. As OpenFOAM has been a widely adopted and often modified tool for a number of engineering communities and computational techniques (e.g. [133, 134]), the potential exists for similar such development for LTP applications as well.

In recent years, improved and easier access to high performance computing resources has contributed to the development of quantum codes for generating cross sectional data relevant to the modelling of LTPs.

### 7.2. Making a case for open-source codes

The description of LTPs often involves solving multidimensional multiscale nonlinear problems, which requires considerable investment to develop incredibly complex codes, some mentioned in the previous section. This is a highly demanding scenario. Considering that it has been estimated that software contains approximately 1–10 errors per thousand lines of code [135-137], methods are needed to alleviate the developers’ work and improve the quality of the final product.

Adopting a review process during the development of research software facilitates debugging and verification activities (tasks which are often underestimated in their scope), contributing to raising code quality and correctness. A review process also brings the additional advantages of providing continuity of the research work and in improved knowledge transfer within and between research groups. The benefits of a code review process to enhance the quality of the scientific work are probably better understood as the reader tries to answer the following questions: Could you rewrite the same code built some years ago, reproducing the same computational results that were then published? Can you easily find the old version of the code used for that publication? How many versions of the software can you find simultaneously being used in your research group? Do you know the differences between these versions? What is the best/most recent version to distribute to a new student? Is there any related documentation? Are you confident about the set of input data to use in the simulations?

A single reader may have answered no to several of these questions concerning their own code and their own research group. The situation is likely not better at the community level. The introduction of a code review process at the research group level could significantly contribute to elevating research standards. By extension, quality would also likely be improved by making the codes publicly available, ideally sharing the source code, verifying its correctness, and evolving the software as part of a collective effort. Obviously, some codes have specialized requirements and should be developed with less openness. However, in many other cases, a shared code is an asset for the community when it comes to transparent reporting on models and procedures.

The standards proposed for any kind of scientific result should also apply to codes. For open-source codes, the review process would naturally take place during software development, at least involving close colleagues within the same research group. However, in all cases verification should be encouraged as part of peer review before publication of the computational results. Detailed information on verification process should be included in reporting (or as a minimum as part of the documentation) to facilitate confirmation of the reported findings (see section 6).

Focusing on software alone is not enough since codes need data to produce results. There seems to be wide acceptance about the need for proper data management, one method being adopting the FAIR principles [58]. This acceptance is demonstrated by an increased activity on the FAIR topic within the LTP community, where the focus is no longer solely on the data itself but also on exchange formats and web-based dissemination protocols. This evolution deepens the premise that the conduit between codes and data (or databases) should ideally be compatible between different codes and databases. Section 4 addresses the standardization of data representation and handling, a first step to ensure interoperability and reusability of codes and data.

However, future progress could go well beyond these first steps, including collaborative development of a collection of open-source ‘foundational libraries’ that could provide the basis for software developers to build computational tools for solving specific problems. Examples of existing collaborative frameworks close to our community are the open-source CFD software OpenFOAM [132] and the free software program Basilisk [138] for the solution of partial differential equations on adaptive Cartesian meshes. An additional advantage in adopting this type of framework is that they can be used as ‘docker containers’. These are open-source platforms that help a user to package an application and all its dependencies (including libraries, post-processing tools) into a container for the development and deployment of the software, thus freezing the version dependencies of the entire computational workflow.

There are impressive advances being made using open-source modules, originally intended for other purposes, to develop LTP modeling platforms. SOMAFOAM, built on the OpenFOAM platform, has enabled computationally scalable, two- and three-dimensional simulations of low and high pressure LTPs [6]. Several open-source plasma models have been implemented using the MOOSE framework [126-130].

The LTP community has been slow to adopt many of these practices. The reasons are due to a combination of factors. The lack of a common programming language, architecture or application dependent codes, restrictions by government and industry on distribution of codes funded by those sponsors, and lack of funding (and enormous effort) to update or rewrite codes are all barriers that would need to be overcome. Software development for engineering physics is often difficult to get funded and so research projects tend to focus on the final outcomes rather than the building of tools to reach these outcomes; in the short term.

**Figure 6.**
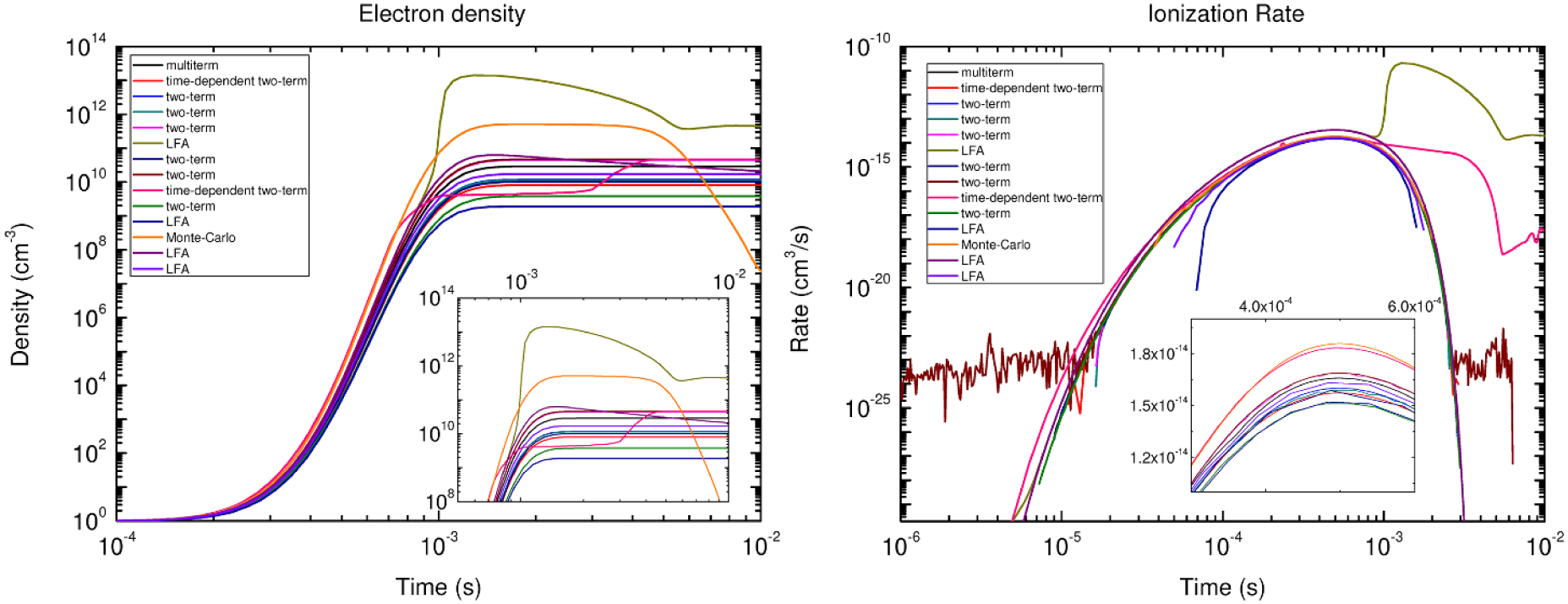
(Left) Time evolution of the electron density and (right) the ionization rate calculated by the participants of the 2017–2018 round-robin exercise for the modelling of a pure argon plasma with four species (Ar, Ar* and Ar^+^ and *e*) undergoing the following electron-impact collisions: elastic scattering with Ar, direct excitation, direct ionization and dielectronic recombination. The plasma is excited by applying an electric field pulse to the neutral gas at 0.1 bar pressure and 300 K temperature, for initial electron and ion densities of 1 cm^−3^. The insert caption identifies the different model approximations that were used, where LFA refers to the ‘local field approximation’ in the Boltzmann-chemistry coupling.

The LXCat workshop field at the 2016 GEC [139], identified the need for a community wide activity on validation of plasma chemical kinetics in commonly used gases, and proposed a round-robin exercise to assess the consistency in results of calculations from different participants in a simplified system. Subsequently, two rounds of exercises were attempted, in some cases revealing unanticipated disagreements in the computational calculations presented by the participants (see figure 6).

Although these differing results can in part be attributed to ill-definition of the detailed working conditions, there was also evidence of (a) different implementations of the same publicly available code and (b) different interpretations or deployments of the physical models and the corresponding input data for in-house codes. Both had an impact on the results (see also section 4). The sharing of codes or, at least, of the details about the numerical implementation of models (including algorithms, convergence criteria, closure/boundary conditions), could significantly improve the quality of computational predictions in LTPs. It could also nurture and support a new generation of researchers developing computational algorithms and models, a population that has continuously decreased in the last decade [140].

### 7.3. Recommendations for code development

**Figure 7.**
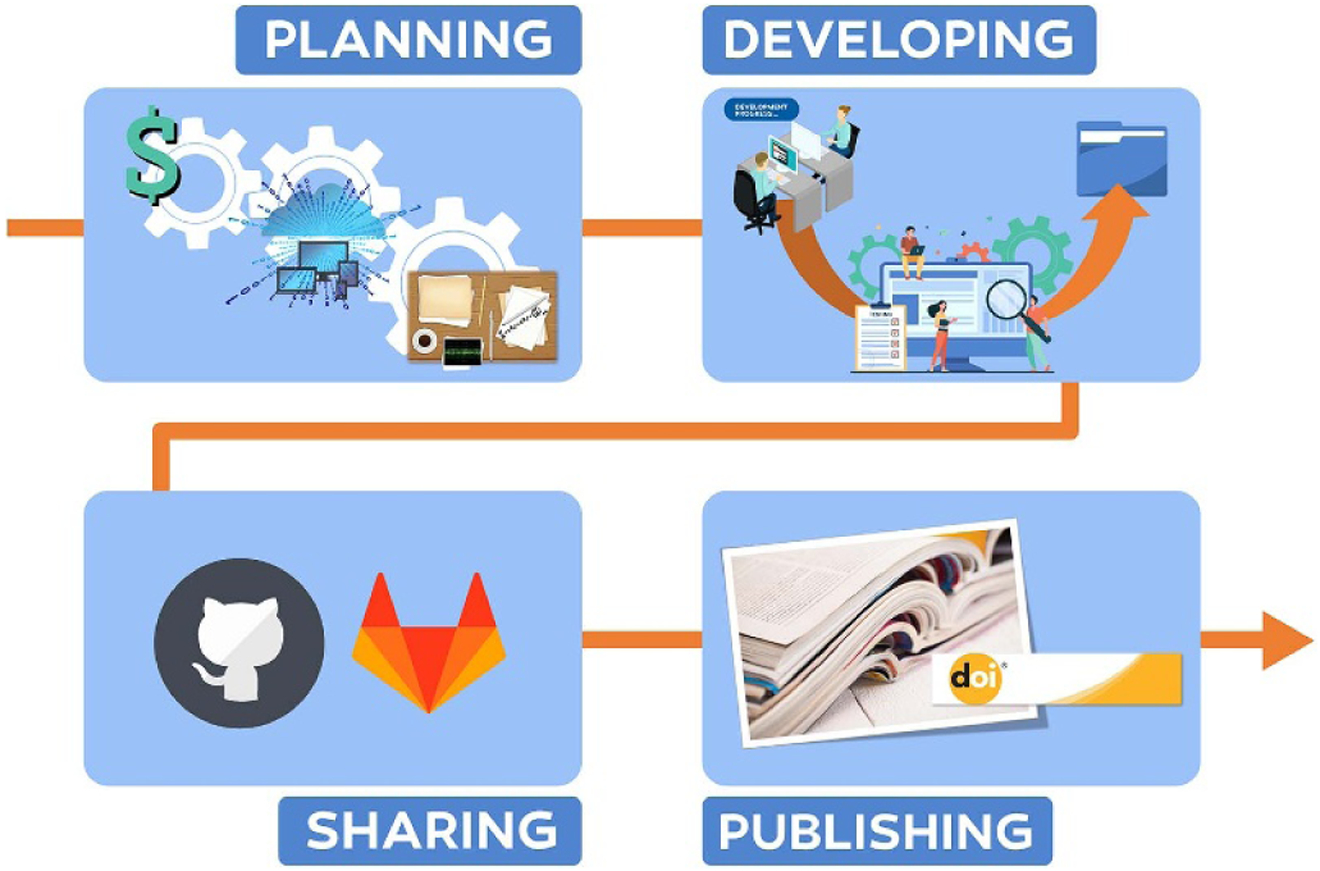
Schematic of recommendations for code development. Designed using resources from Freepik.com.

In the following we provide some practical recommendations (see figure 7) for developing research software that is open and adheres to FAIR principles [141, 142].

#### 7.3.1. Planning.

As in any other research activity, planning is essential for an effective and successful outcome. In the case of software, this involves several aspects: financial (securing the necessary resources for the task), technical (deciding about the programming language to adopt), and scientific (formalizing the problem, preparing the numerical implementation and choosing numerical methods and algorithms). Ensuring the compatibility of the software with I/O databases and platforms relevant for the community should also be considered during the planning phase.

#### 7.3.2. Developing, reviewing and verifying.

Introducing a review process during code development should contribute to ensuring code correctness and enhancing the quality of the scientific work. Code review implemented as a discussion thread (under some versioning system like Git [143] or Mercurial [144]), with the ability to comment and suggest code changes, can work toward improving debugging outcomes, preserving code legacy, and providing a natural distribution of the workload during both development and maintenance phases.

Code review should also include verification procedures (e.g. checking that the code reproduces asymptotic limits, satisfies the conservation of quantities, and provides results similar to other codes, within numerical uncertainties) and regression tests (to confirm that new code changes do not affect the existing functionalities). More details and examples of the verification process are discussed in section 6.

#### 7.3.3. Sharing.

Making codes publicly available is the next step toward software adhering to FAIR principles. Publishing the code on the research group website or, for open-source codes, making it accessible on source-code-repository-hosting platforms such as GitHub [145] or GitLab [146], should, when allowed by sponsors and government agencies, become a standard practice for research software of interest to the community at large.

#### 7.3.4. Publishing.

Like any other research result, codes should also be published in a journal that accepts software as primary submission material [147]. Moreover, even if the code is available in a public repository and because these web resources might not be permanent, assigning a digital object identifier to the version referenced in a published manuscript is also highly recommended. This must be done carefully so that updates and corrections to codes can be tracked and made available to the community.

### 7.4. Outlook

Many of the recommendations discussed in this section are not new. Indeed some were raised decades ago [148]. However, implementing the previous recommendations is a challenging task that requires a culture change from researchers, publishers and funding institutions, where openness is supported and sharing software is encouraged as part of the high-quality standards in scientific research. The LTP community should use the main conferences in the field as a forum to design and carry out collective efforts for the verification and benchmarking of codes, following the recommendations listed in section 7.3, for developing research software.

## 8. Reporting

Historically, reporting of data, how it has been analyzed, and how it is presented has been heterogeneous for the LTP community. Most journals do not have specific standards on how to report data, and when there are standards, they often vary from journal to journal. In the spirit of a research enterprise that collectively advances our scientific knowledge and understanding of how the world works and the technology that drives it, accurately reporting data in journal articles is essential. Accurately reporting data (a) makes clear the underlying information, and how it was acquired, that leads to scientific conclusions, (b) enables readers to assess the validity or quality of that information, and (c) empowers researchers to reproduce the results to confirm said conclusions. From this perspective, reporting of data necessarily has significant overlap with data availability, but takes it one step further to also include how the data is analyzed, assessed, and presented in the published literature. For this reason, reporting of data also often intersects with issues surrounding ethics and scientific misconduct.

### 8.1. Defining reporting and how it applies to plasma research

**Figure 8.**
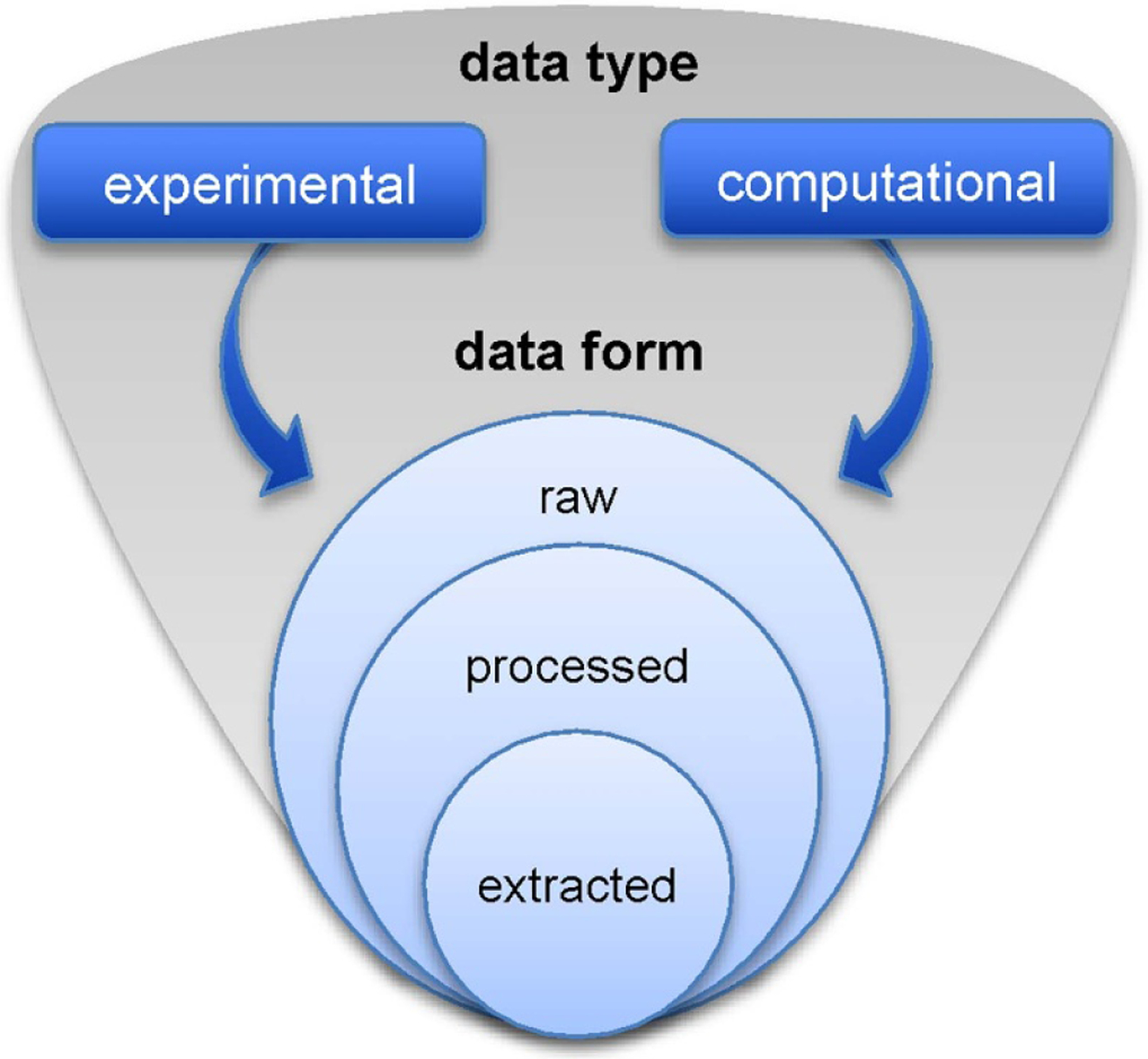
Different types of data and their sources and the different forms in which they are reported.

Here, we define reporting of data to broadly mean how data is presented in published archival journal articles, including not only the main text but any accompanying appendices, supporting information, or supplemental material. Here we emphasize the standards on reporting of data. Standards for the dissemination of data are discussed in section 4. Data is usually presented in one of two ways—graphically or in tables—but it can also be reported as individual values within the text of an article. Furthermore, the concept of data is vast, and data itself can encompass almost innumerable forms. Here we classify data into two general types, experimental and computational, and in three general forms, raw, processed (or analyzed) and a third category we term extracted data, as shown in figure 8.

**Figure 9.**
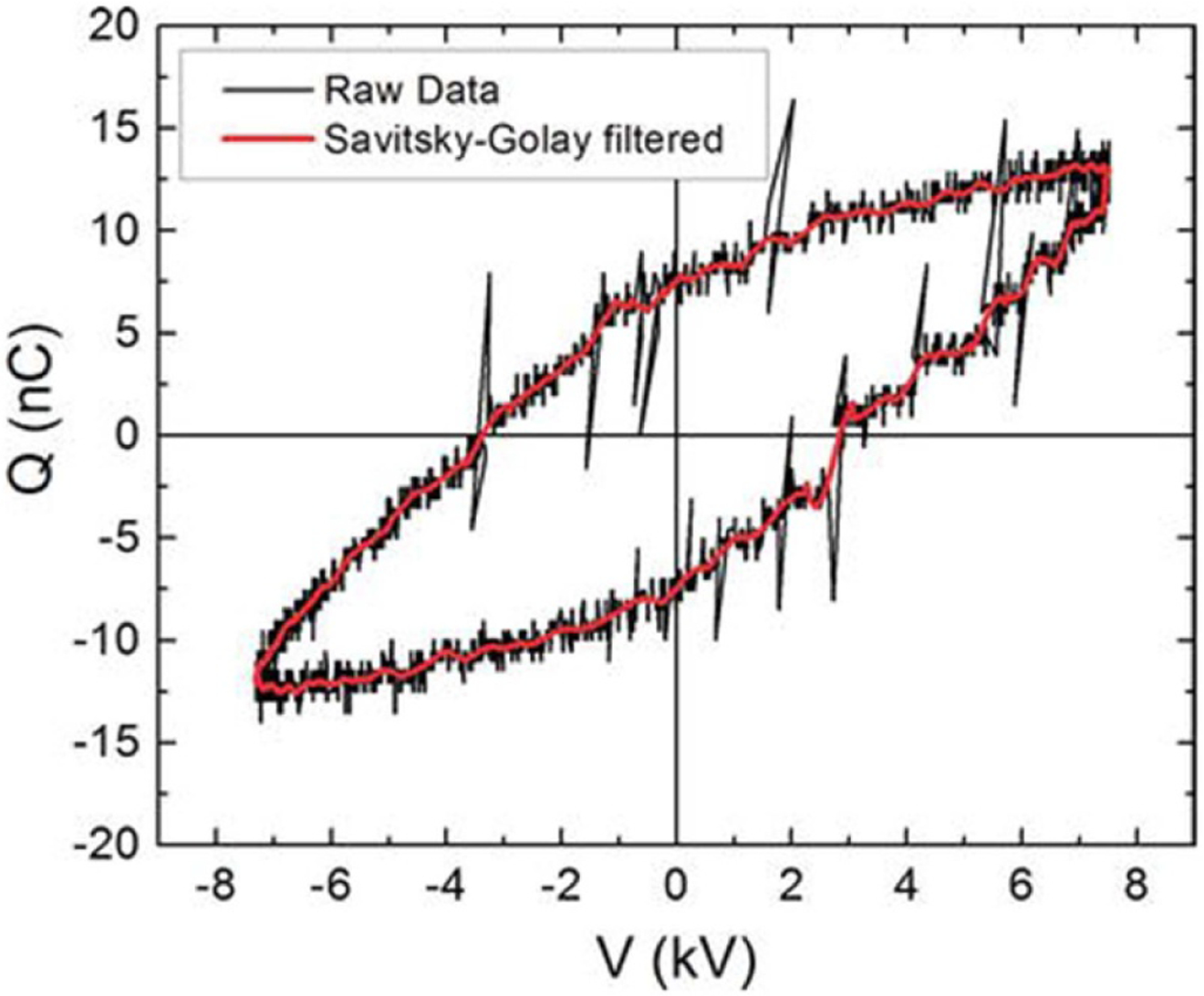
Example of filtered charge-voltage data, also known as a Lissajous plot, from a dielectric barrier discharge. Reproduced from [149]. CC BY 3.0.

The differences between experimental and computational data are fairly straightforward. Was the information generated from a physical process itself or by virtue of numerically solving mathematical descriptions of the physical process? The differences between raw and processed data are similarly straightforward. The raw data are the data as produced by the data acquisition equipment. Processed data changes the raw data to make it more easily readable or interpretable such as filtering spurious or low signal-to-noise-ratio data. For example, a spectrum produced via OES will include line intensities on an absolute scale related to the number of photon counts by the detector—this is raw data. Manipulating the data to normalize it or subtract the baseline noise, would be characterized as processed data. In electrical DBD measurements, the raw current data may include the capacitive current, but it is often presented ‘processed’ to only include the discharge conduction current. Similarly, raw charge-voltage data used to produce a Lissajous plot may be smoothed by a filter, as illustrated in figure 9. Computational data can also be processed. For example, in particle kinetic simulations, such PIC Monte Carlo collision simulations, raw calculated data is often averaged over several integration-times in order to suppress the random statistics inherent to the method.

Both experimental and computational data can also be further analyzed to extract physical quantities, which adds another layer to data reporting. For example, OES spectra can be analyzed to extract characteristic plasma properties including electron temperature, electron density, and relevant excited state temperatures [150-152]. Similarly, DBD measurements can be analyzed to extract the power deposited in the discharge [149, 153] or parameters such as the number of filaments per cycle [154]. Within the context of computational data, the governing equations are typically solved numerically for quantities including energy or velocity distribution functions, species concentrations, momenta, and energies, electric potential, and magnetic and electric fields. However, other parameters can also be extracted from the computed data, such as transport coefficients and production/destruction rates from computed distributions [155], and ionization and chemical conversion efficiency from the computed concentrations [156]. Critically, depending on the model adopted in constructing the governing equations, additional processing might be needed to extract physical quantities.

### 8.2. Uncertainty, error, and how it relates to reporting of data

An essential element of reporting data—both experimental and computational—is clearly explaining the confidence that one has in the data, where confidence is defined here as the statistical probability that a certain fraction of the same experiments or computations would produce the same data. Conventionally, the confidence in the data is related to how accurate it is relative to the *true* value that exists independent of the measurement [157]. For experimental data, reporting uncertainties based on both precision (or repeatability of experiments) and bias (inherent accuracy of the measurement system, instruments, and method) effects is typically expected, whereas for extracted data, uncertainty propagation analysis should be used. Further, for small data sets, Student’s *t*-analysis should also be used. On the computational side, accuracy of data is related to both the verification and validation of the computational code [158] as discussed in section 6, with emerging techniques also focusing on incorporating uncertainty quantification into computational simulations [159]. Both areas have begun to receive appreciable attention in the LTP community [100, 160, 161].

### 8.3. Emerging trends and standards in the broader research community

Over the past decades, a number of research communities have raised concerns about standardizing how their data are reported. In the life and social sciences, there has been significant focus on and momentum toward more effective and accurate data reporting in an effort to mitigate intentional and unintentional shading of the results to support a particular outcome, to produce more reproducible and replicable research, and to eliminate bias in the scientific method [162-164]. Physical sciences, such as chemistry and physics, have also seen an increasing trend in this heightened awareness [165-170], such that collectively it has become apparent that the entire scientific community is moving toward more openness and transparency in data [171-173]. FAIR data principles, where the four principles are findability, accessibility, interoperability, and reusability, have, in particular, started to attract widespread endorsement and acceptance [58]. An initial draft of a reporting standard for data in the field of LTP has been introduced with the plasma metadata schema (MDS), plasma-MDS [44], which aims to support the implementation of the FAIR data principles.

**Figure 10.**
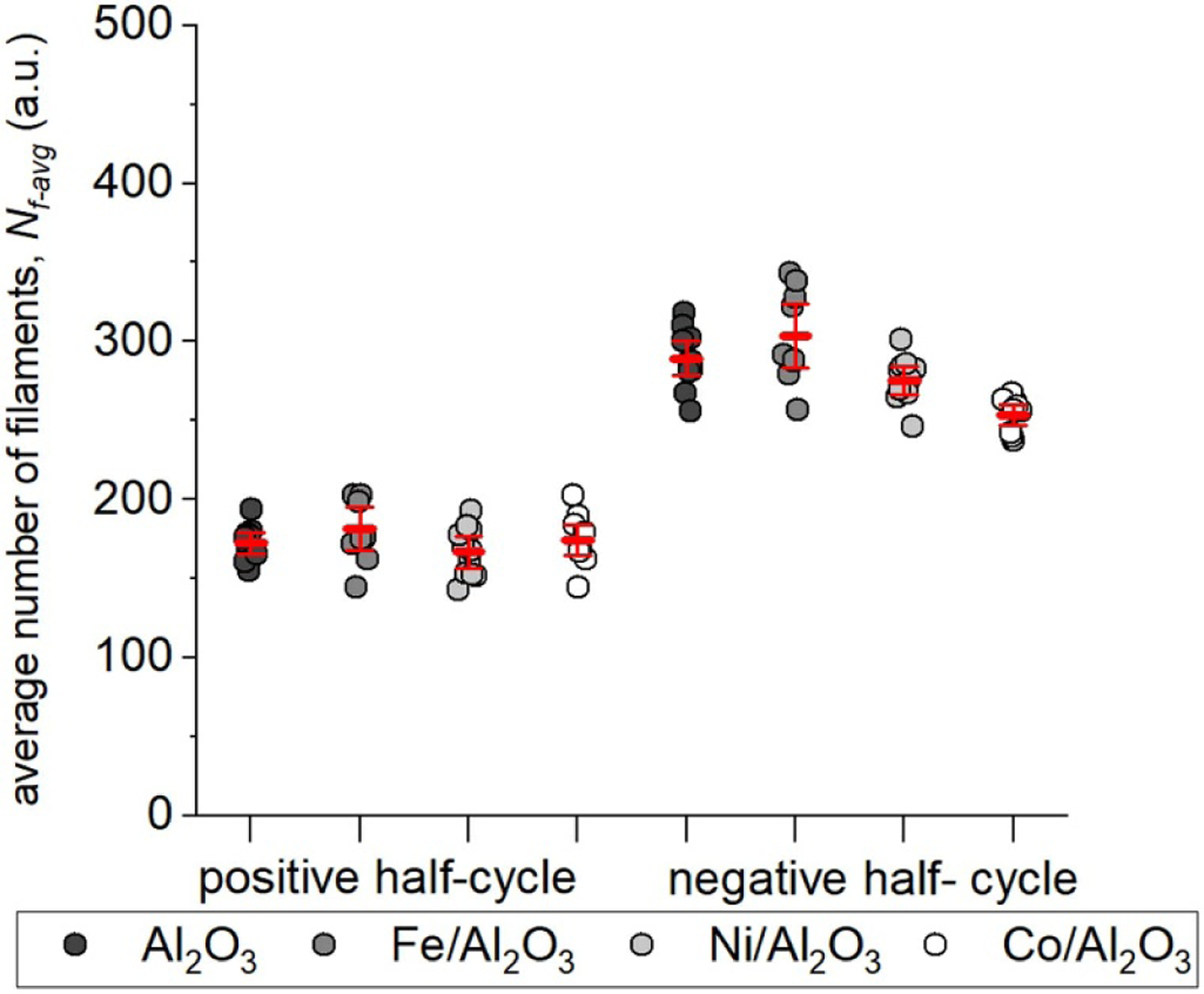
Example of data being presented as the raw data (points) with the statistical information overlaid (red bars for the respective averages and 95% confidence interval error bars). Data are from DBD experiments in packed beds with different materials and reflect the average number of filaments per half-cycle for different material configurations. Reproduced from [154]. © IOP Publishing Ltd. All rights reserved.

Depending on the nature of the research and the types of data that are being reported, different concerns arise. For example, in the life and social sciences, statistical analysis is paramount, and thus ensuring the statistics and data are presented well is essential. There has been a noticeable trend in the graphical presentation of data to move away from simpler *x–y scatter* plots, which show single points with error bars (or confidence internals) that are often not well-defined and ambiguously open to interpretation, to scatter plots that include the raw data with the statistical information overlaid [174]. (See, for example, figure 10.) In the physical sciences, concerns often arise based on how extracted quantities are determined and presented, where inconsistencies within a field make it difficult to both assess the validity of the claims in the paper and to compare to prior literature. For example, in areas such as photocatalysis [167] and photovoltaics [175, 176], accurately determining and reporting parameters such as conversion efficiency and stability of new materials or designs is fraught with difficulties. Given that there are presently no standards for how these quantities should be defined, it is that much more important that reporting make clear the method that was used.

While a number of editorials have been written over the past decade [177], most scientific journals do not have explicit standards on reporting of data within their papers. While policies on availability of data, such as the Institute of Physics (IOP) Publishing data availability policy [178] that governs *Plasma Sources Science and Technology*, are common for many journals, few have policies on how data must be presented and/or requirements on providing information on the nature of the data and how uncertainty was analyzed. For example, the IOP policy includes unbinding rules such as ‘Authors are encouraged to share their data but not required to. If your data will not be made publicly available, the journal requires that any data required to support or replicate claims made in an article should be made available to the journal’s editors, reviewers and readers without undue restriction.’ and ‘Sharing research data as supplementary information files is discouraged’ [178].

There are exceptions, though. The *Science* family of journals has a set of ‘Research standards’ as a part of their editorial policies, and these include specific guidance on statistical analysis and how data are reported [179]. The level of detail is greater than the norm, with two examples of policies being:

- ‘Data pre-processing steps such as transformations, recoding, re-scaling, normalization, truncation, and handling of below detectable level readings and outliers should be fully described; any removal or modification of data values must be fully acknowledged and justified.’
- ‘Methods used for conducting statistical tests (e.g. *t*-test, Wilcoxon signed rank test, Wald test of regression coefficient) and for constructing confidence intervals (e.g. normal-based 95% confidence interval: mean 2SD, likelihood ratio-based interval) should be clearly stated. Mention methods used in the Materials and methods and then provide the individual test name in the figure legend for each experiment.’

Similarly, the American Institute of Aeronautics and Astronautics (AIAA) family of journals has an editorial policy that specifies what is expected in demonstrating both experimental and numerical accuracy when reporting data [180]. However, as noted in other fields, editorials and editorial policies do not always have the desired improvement in reporting [181-183]. To overcome the limited effectiveness, some publishers, with *Nature* being the most prominent, require authors to provide detailed information that affects data reporting in the form of checklists that must be included at the time of journal submission [184], with more stringent requirements for specific areas such as photovoltaics [185] and reports of lasing [186].

### 8.4. Opportunities and challenges for the plasma science community

The plasma science community as a whole is incredibly heterogeneous, with the low-temperature, non-equilibrium (non-thermal) community quite distinct from the high-energy and density (thermal) community. Even if just narrowing to LTPs, the types of data are incredibly vast because the field itself is so diverse. With much of current LTP development being application-oriented, data is often focused on a specific application. Even considering measuring only classical plasma properties, there is a wide variety of intrusive and non-intrusive electrical and optical diagnostics that could be used (as discussed in section 3). It would be challenging to define standards on data reporting for all but a few of the most widely used techniques (e.g. OES or Langmuir probes). As many of the desired quantities are not measured directly and require extracting information from directly measured data, it is difficult to develop standards that encompass the entire field. A good example of this, for example, is the measurement of delivery of reactive species to a substrate, where the community has not formed a consensus for the definition of ‘dose’, as discussed in section 9.

Computational efforts have similar challenges, as many different research groups utilize their own in-house codes, whether those be particle- or fluid-based. There is no clear roadmap for the verification and benchmarking of these codes, and no consensus on how to validate these codes against an experimental standard, although recently there have been efforts to standardize these [61, 94, 96, 187, 188] (see section 6 for more information). Given it is clear that a one-size-fits-all set of reporting standards is not possible, it is instructive to look where there are opportunities to standardize reporting for the LTP community.

The purpose of reporting new research findings is to *advance our collective understanding and knowledge about natural and physical phenomena.* Essential to this goal is the ability for others to confirm reported findings. Reporting data (as supplementary material, eventually archived in well-established repositories with persistent web links) and how they were *determined* and *analyzed* as transparently as possible should be an essential element of scientific reporting in archival journal articles. Data accessibility recommendations, such as the aforementioned FAIR data principles, are therefore important and complementary to reporting standards.

These principles extend to detailed descriptions of the methods and techniques used, including any experimental instruments that introduce inherent bias uncertainty. For example, if spectroscopy results are presented in any form—either as raw data or results extracted from raw data—then the resolution of the spectrometer, the integration time, the grating, the calibration process, and other pertinent device specifications should be included in the paper. Information about grid resolution, time steps, and convergence criteria in computational simulations similarly should be included. Being explicit about statistical analysis of experimental data, verification of computational simulations, and similar data analysis techniques should be standard in journal articles or supplementary material. Figures and their captions should be presented *unambiguously*. That is, it should be clear what is being presented, in what units, and what the uncertainties are so that the conclusions drawn from the figure are well informed. Ultimately, these recommendations are all in alignment with the principle of making data reusable, and resources such as the plasma metadata schema plasma-MDS [44] that provide a framework for including a wide variety of information should become commonplace in the plasma community.

In areas of plasma science and engineering that overlap with other fields where there are established standards, those standards should be followed. For example, plasma catalysis is a rapidly expanding field that generally applies a plasma to a catalyzed reaction. Existing standards for reporting catalytic performance [166, 189, 190], such as using turnover frequency to report the intrinsic catalyst activity, should be adopted when possible. When those standards need to be modified, such as to distinguish between plasma-only and plasma-catalyst effects [191], that should be done in a way that is transparent and clear to the reader. Similar principles can be applied to other emerging areas such as plasma medicine, plasma agriculture, and plasma combustion.

It is critical to reference and report information from original sources. For example, in many plasma simulation codes—both fluid and particle—energy-dependent collision-cross sections can be extracted from data repository websites, such as LXCat (the Plasma Data Exchange Project) [115]. However, when reporting usage of information, authors should point readers to the *original* source, and not the repository. Apart from the due recognition, the reason is also to ensure findability and accessibility of data, since online material hosted at web addresses have no guarantee of perpetuity. Similar sentiments hold for review articles that collect information but are not the original source of data. Like in several other ethical issues above, the responsibility for complying with guidelines lies with all parties involved—authors, managers of data repositories, journal publishers, editors, and reviewers. Indeed, these obligations should be clearly stated in the rules for referencing of the repositories (e.g. for LXCat [192]), in the policy of the journals about data citation (e.g. for IOP journals [178]), and also in the guidelines for the reviewers [193], who are empowered to scrutinize the material submitted.

The goals of transparency and reproducibility are difficult to argue against, and so the process adopted to achieve these goals cannot be overpowering and burdensome. If so, the process will not be adopted, and these goals will not be met. In the end, judgment may prevail. Some of the most defining discoveries of the LTP field were based on what today we might consider single point measurements made in a single experiment. Being able to accommodate new discoveries and innovation, while being transparent within a system of standards and requirements is vital to the advancement of the field.

## 9. Plasma dose

Plasma dose (PD) is a measure of plasma produced reactivity. PD is a concept that is intended to minimize uncertainties in comparing the results between experiments and laboratories when, for example, treating surfaces with different plasma devices. If the surfaces are treated with the same PD, then in principle the activating species delivered to the surface are the same. With the same activating species delivered to the surface, attention can focus on the consequences of that treatment on the surfaces. There have been several proposed definitions for what PD implies, most of which have originated in plasma medicine [194-198]. These concepts attempt to define PD as the amount of exposure of a biological sample by a particular plasma device to produce a specific biological outcome. For example, PD might define the amount of exposure of an *Escherichia coli* culture to an argon plasma-jet that is required to reduce the viable population by a given amount.

The use of PD for plasma medicine is motivated by the use of doses that are standard in radiation chemistry [199-201]. Here, a dose is a given fluence (time integration of flux) of x-rays or a given energy deposition by electron or proton beams. The successful use of dose in radiation chemistry is, in part, a result of the delivery of the activation energy being essentially independent of the final application and of the ambient environment. This independence results from the activation energy—the electron or x-ray beam—being generated by a stand-alone accelerator with there being essentially no feedback from the object being treated. The use of dose in radiation chemistry is typically separate from an assessment of the outcome of using that dose.

There is a tendency in plasma-biomedical applications to associate PD with a particular biological outcome. In spite of its utility, defining PD in terms of a biological outcome is perhaps too high-a-bar to pass in defining dose, at least at this early stage of discussion. Such a definition would require consistency and standards in how the sample is prepared (initial density of colony-forming-units, type and depth of culture medium), the geometry of the setup (plasma source-to-sample distance, gas residence time, vortexing or recirculation of gas), environmental factors (ambient humidity and sample temperature) and method of measuring surviving cells, to name only a few. Having a standard that describes all of these properties for the purpose of calibrating a protocol or set of equipment would be extremely helpful. However, that is a different issue than PD.

In the context of this discussion, we describe PD in less ambitious terms by not associating PD with a particular outcome. Doing so would tie PD to a specific application which then would require additional standards for how that application is configured. To be as universal as possible, PD should be as independent of the final application as possible, in analogy to the practice in radiation chemistry.

For the purpose of this discussion, PD is defined as the net fluence (time integral of flux) of a reactive species (or sum of species) delivered by a plasma device through a conceptual surface. The PD would then be defined for individual species [Ar ions, PD(Ar^+^); or ozone, PD(O_3_),] or collectively for a set of species [reactive oxygen species PD(ROS); or reactive nitrogen species PD(RNS)]. PD requires units and so for the purpose of discussion, we define the unit of PD as being the fluence that would expose, on the average, each site on a surface to one reactive species. With the surface site density approximately 10^15^ cm^−2^. This could be achieved by delivering a flux of 10^15^ cm^−2^ s^−1^ for 1 s, or a flux of 10^14^ cm^−2^ for 10 s. So for this discussion, 1 PDu(O_3_) is delivery of 10^15^ cm^−2^ O_3_ molecules over a specified period of time.

**Figure 11.**
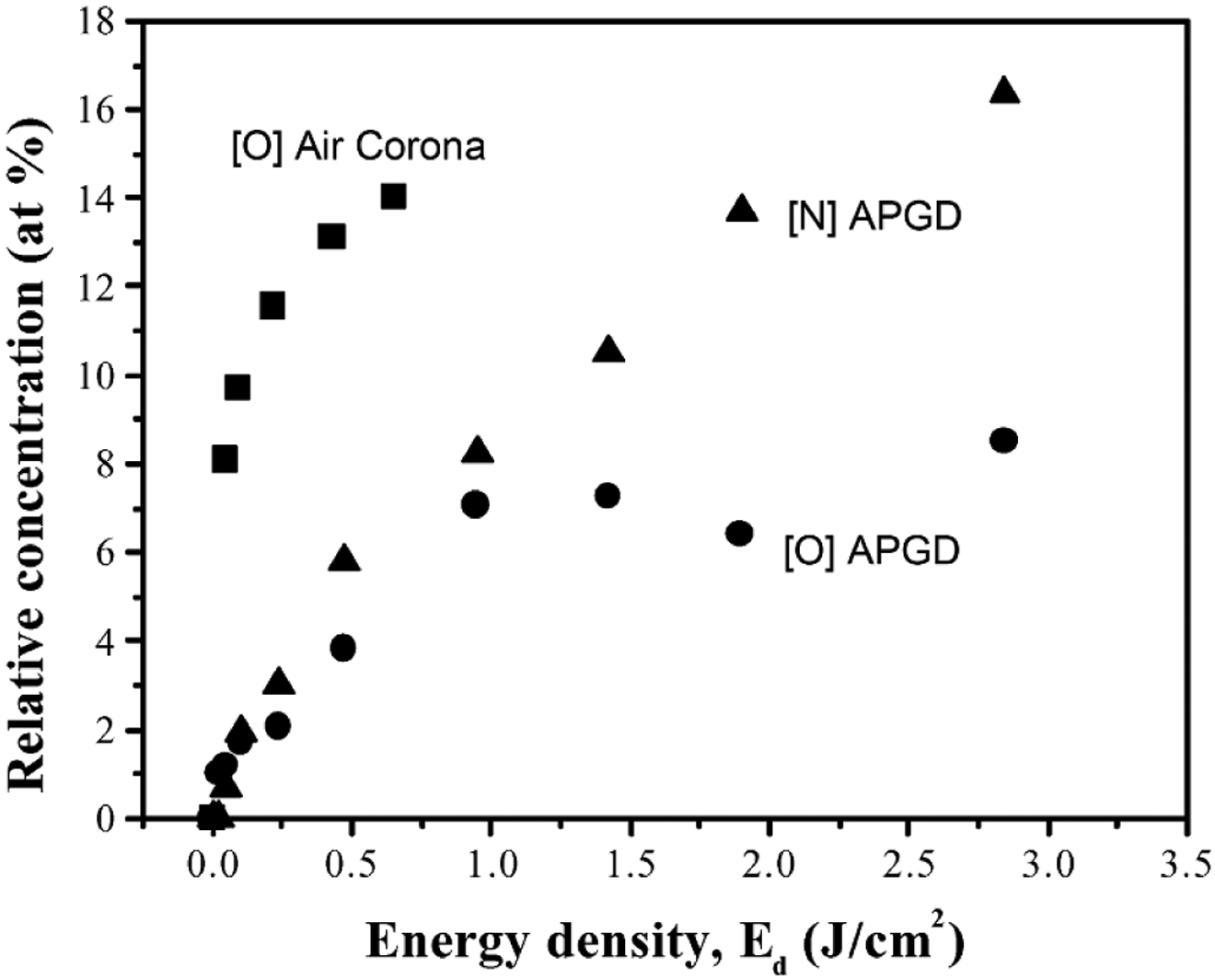
Surface concentrations of oxygen [O] and nitrogen [N] as a function of J cm^−2^ of plasma energy incident onto the surface after treating biaxially-oriented polypropylene (BOPP) using an air corona and an atmospheric pressure glow discharge (APGD) sustained in nitrogen. The repetition rate was 1 kHz. Reproduced from [202], with permission from Springer Nature.

The concept of a fluence-based dose is already implemented in plasma surface treatment of polymers. For this application, treatment is characterized by the J cm^−2^ delivered to the surface [203, 204] although the range of operating conditions for which this dose-driven description applies is limited. (See figure 11.) Characterizing treatment of polymers by J cm^−2^ is typically done for atmospheric pressure plasma treatment in a specified ambient—for example, air or He. We also note that in plasma-based chemical conversion and the emerging field of plasma-catalysis, there is also a concept of energy delivery, which is termed the specific energy input and is defined as the amount of energy deposited by the plasma per volume or molecule of gas, typically expressed in J cm^−3^, kJ L^−1^, or eV/molecule [205]. This measure of plasma activity is distinct from the concept of dose, as it reflects the excitation of the plasma molecules themselves as opposed to the fluence of excitation delivered by a plasma to a surface.

The recent discussion of PD originated in the context of atmospheric pressure plasmas for treating biological systems. At atmospheric pressure, advection typically dominates over diffusion, and so the directionality of the flux constituting the dose is more easily defined at atmospheric pressure (again, in analogy to radiation chemistry) as opposed to low pressure where diffusion dominates. However, in principle, PD is (or should be) independent of pressure, flow-rate or other ancillary conditions—*fluence is fluence*. In spite of the universality of fluence, it will be necessary for plasma device manufacturers to be very explicit in how they define their device’s capabilities in terms of delivering PDu. Such a specification for a plasma jet might be—the plasma device will deliver 10 PDu of O_2_(^1^Δ) every 20 s through a 2 mm × 2 mm window centered on axis and located 1 cm from the orifice of the device when using the standard operating conditions. Similar specificity would be required by researchers in reporting the outcome of exposure of a surface by a given PDu of O_2_(^1^Δ).

Such descriptions of PD by manufacturers or researchers say nothing about what else might be delivered along with the specified PDu(O_2_(^1^Δ)). Doses of ions, photons or reactive nitrogen species may be delivered along with the PDu(O_2_(^1^Δ)). These other doses may affect the final outcome of treatment, but do not affect the fact that the plasma device delivers a particular PDu(O_2_(^1^Δ)). A specified PDu of O_2_(^1^Δ) at 1 cm from an orifice also says nothing about the PDu(O_2_(^1^Δ)) at 0.9 cm or 1.1 cm from the orifice.

It is true that a PDu(X) likely varies within a given system, which then requires care in where the PDu(X) applies. For example, a flowing low pressure plasma delivering 10 PDu(O) at 1 cm above a surface likely also delivers nearly the same 10 PDu(O) at 0.9 cm and 1.1 cm above the surface. The rate of gas phase reactions of O atoms is low at low pressure and so the flux of O atoms would likely be nearly the same at all of these locations. At atmospheric pressure, the gradient in O atoms is much steeper due to the increased rate of reaction of O atoms. As a result, PDu(O) at 1 cm would likely be different than at 0.9 cm and 1.1 cm, and could be very different.

This working definition of PDu implies some aspect of linearity of the outcome of the delivered dose, which is typically the case in radiation chemistry and polymer treatment. The same PDu can be achieved with different combinations of convective speed, density and exposure time. A dose of 10 PDu(O_2_(^1^Δ)) over 10 s with a convective speed of 10^3^ cm s^−1^ corresponds to a density of O_2_(^1^Δ) of 10^12^ cm^−3^. Ten PDu(O_2_(^1^Δ)) delivered over 1 s with a convective speed of 10^2^ cm s^−1^ corresponds to a density of O_2_(^1^Δ) that is 100 times larger. If there are non-linear aspects of the plasma chemistry or surface treatment, the same 10 PDu(O_2_(^1^Δ)) delivered for these two conditions might result in different outcomes. These potentially different outcomes emphasize that to have any utility, PD should not be tied to outcomes. PD would best be conceived as a measure of delivery of a given radical, ion or photon.

Condition dependent outcomes while keeping a scaling parameter constant are not unique to PDu. Consider one of the most fundamental scaling parameters in LTP science—*E/N*, electric field/gas number density, measured in terms of Townsends (1 Td = 10^−17^ V cm^2^). Operating two devices with the same *E/N* only implies that the rate coefficients for electron impact processes of the ground state will be the same (assuming conditions akin to the local field approximation). However, the outcome of those identical rate coefficients could be very different for systems operating with the same *E/N*. Consider operating an oxygen plasma at 10^4^ V cm^−1^ at 1000 Torr compared to 1 V cm^−1^ at 0.1 Torr. The outcome of exciting, dissociating or ionizing an oxygen molecule at the same *E/N* will be very different in these two systems. Even for the same pressure and same *E/N*, the outcome would be different for different current densities.

At the time that the concept of *E/N* was first proposed, there was also an understanding of the limits of describing a system using *E/N*. Those limits include that excitation must be dominantly by electron impact from the ground state, partial ionization should be small to reduce the influence of electron–electron collisions, gradients in density should be small compared to the distance over which the measurements are made and the time for electrons to come into equilibration with the applied electric field should be short compared to the time of interest. Today, experiments often violate the basic assumptions that enable comparing systems operating at the same *E/N*. For example, the purpose of ns-pulsed plasmas is to overcome these very limitations. Nevertheless, *E/N* remains a hugely valuable method of comparing plasma systems, even if the precision of those comparisons is not high.

A manufacturer specifying that a device delivers a given PDu(O) or a researcher reporting that treatment of a surface was performed with a given PDu(O) requires that the measurements of O densities and convective speeds be properly performed. Having said that, PD should be independent of the manner of measuring PD. Verifying that the measurements have been done properly is discussed in section 3. It should also be recognized that measuring dose is by no means easy and for some species, there is no consensus on the best approach to do so. Benedikt *et al*, for example, recently outlined some of the best approaches for measuring flux, but also some of the continuing challenges [206]. The entire plasma community would greatly benefit from advances in measurement techniques for quantifying dose of different particles and converging toward accepted standards.

The concept of PD is very appealing and, in some ways, is necessary to promulgate the use of plasmas to non-plasma experts. Such examples come from the biomedical radiation chemistry community. Patients are treated by electron-, proton- and x-ray beams based on delivering a dose (e.g., J cm^−3^) while the practitioners are not necessarily experts in the technology delivering those doses. For this reason alone, standards for PD (and PDu to measure that dose) should be developed that are independent of the final application. However, the expectation that PD can be used to precisely compare LTP systems may be too great a task for today. It may be better to think of PD in the same manner as *E/N*—a concept that strictly applies to idealized systems and which becomes less precise as those systems diverge from those idealized conditions. PD would nevertheless retain high value in spite of the lack of precision, just like *E/N*, in providing guidance, design principles and enabling initial comparison between systems.

## 10. Technology transfer

**Figure 12.**
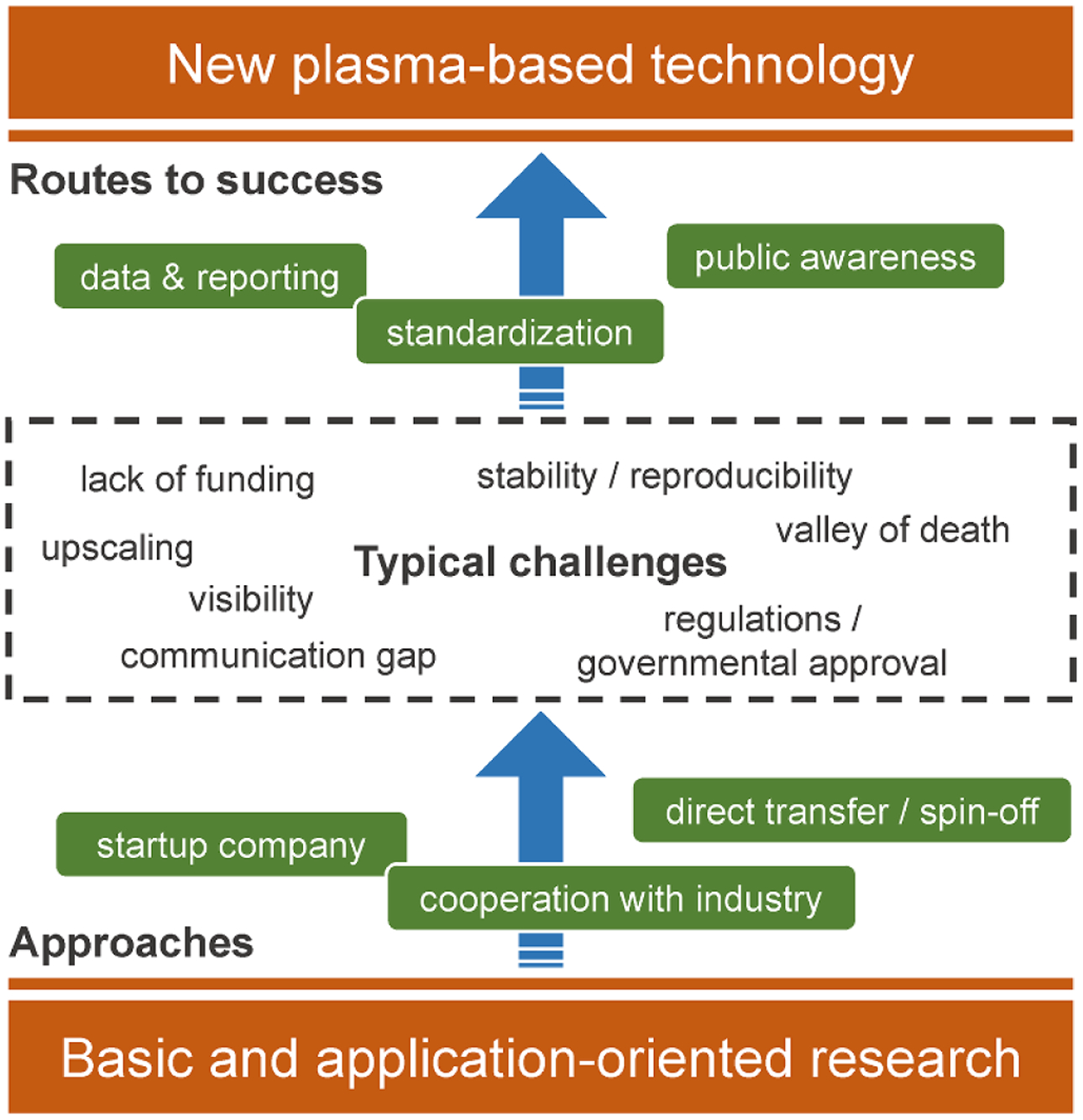
Common approaches, typical challenges and identified routes to success for technology transfer in the field of plasma science and technology.

This section addresses the transfer of research and development results to practical applications. The transfer of basic research to technological applications has long played a major role in LTP physics, with an early example being technology invented by Siemens in 1857 for ozone generation by means of silent discharges. Nevertheless, technology transfer in this research area still faces barriers that often prevent or slow the direct use of research results for establishing new technologies. These barriers are different in the various applications of LTPs in industry and the healthcare field. These challenges, common practices and requirements in transferring (or translating) experimental techniques, devices and codes to specific applications are discussed here. Methods are suggested for how technology transfer can be supported and efficiently organized in the future. (See figure 12.)

Basic research takes place primarily at universities and national laboratories, which face particular challenges in the transfer of fundamental knowledge. University technology transfer in LTPs face problems similar to other fields of knowledge, with some accrued difficulties in identifying the relevant marketplace for applications that are often at the intersection of several disciplines. These issues strongly depend on the specific technology, and the perspectives and outcomes can vary by country or region (US, Europe, Asia) due to cultural, organizational and financial reasons. In spite of these differences, university technology transfer typically follows common standards and comparable challenges.

- Modifying the desired outcomes of the research plan in terms of moving from fundamental-oriented to application-oriented, transforming the research results into a ‘product’ with commercial added-value, and perhaps redefining scientific output with patents replacing papers in scientific journals;
- Funding expectations, in terms of the initial investment needed to launch a startup company, and/or to develop a prototype that can be commercialized with a higher technology readiness level that satisfies possible regulatory requirements;
- Maintaining the patent or the portfolio of patents that protects the invention;
- Making decisions about the best route for the commercial exploitation of the invention, concerning both the management of the intellectual property (opting for a controlled disclosure under a patented protection or using confidentiality agreements), and its exploitation (under a startup company with the participation of the inventors, by licensing the research product at certain royalty rates, or by selling the invention preferably keeping access to the evolution of knowledge).

In general, technology transfer works particularly well when industrial partners are involved in research and development from the outset. This is common practice in material and surface processing industries, where plasma technology has been well established for decades [207]. However, bridging the gap between laboratory scale solutions and industrial requirements remains challenging. Reproducibility of plasma processes and plasma uniformity on large scales are among the issues in this regard and extensive consulting work occurs after transferring technologies if devices do not work as they were originally expected. This challenge can be heightened by lack of full disclosure. Proprietary knowledge is often not fully shared even if non-disclosure agreements are in place. In this respect, long-term partnerships between research institutions and companies are advantageous before the start of a joint development project as a trust relationship has likely already been established. Unlike for medical and biomedical applications, standards and standard operating procedures do not play a particularly critical role in the field of plasma-based materials processing.

Challenges of a completely different kind occur in the healthcare field, where plasma technology is not yet widely deployed, although research has been conducted in this area for more than a decade [195, 208, 209]. The major issue is the governmental approval (e.g. the medical device regulation in Europe), that is required to use plasmas for medical applications. Particular challenges include patient safety with respect to high-voltage security and electromagnetic compatibility, which follows the international standard IEC 60601-1 ‘Medical electrical equipment—Part 1: General requirements for basic safety and essential performance’. Two-fold challenges arise from the fact that plasmas are complex and not (yet) well known by the inspection and certification agencies. On the one hand, it is difficult to introduce a secure and well-tested plasma device for a specific application when the response of the regulator may be ‘what is plasma?’. On the other hand, it might happen that new plasma devices are approved just because the first device also using plasma technology received approval with the regulator assuming that all plasmas are alike.

These difficulties can be overcome by means of testing standards. One attempt to unify requirements for plasma medical devices has been made by introducing the DIN SPEC (Deutsches Institute fur Normung specification) 91315 [210]. National and international standards are consensus-based and should be developed with the participation of all stakeholders. As the field of plasma medicine is still relatively young, the DIN SPEC was chosen as a first attempt, which is a special standardization defined as specification, and can be developed by a temporarily appointed committee advised by DIN eV. (Berlin, Germany). The DIN SPEC ‘General requirements for medical plasma sources’ describes basic criteria for the characterization of medical plasma devices, referring to international or national standards whenever appropriate, and creates a basis for physical and biological characterization of plasma and its effect on cells.

The DIN SPEC was first published in June 2014 with the intent of developing it further into a DIN/international standard with community consensus. Although that has yet to be accomplished, multiple studies have been published following the DIN SPEC protocols [211-215]. With a set of standard characterization ranging from gas temperature of the plasma to patient leakage current, inactivation of defined microorganisms and viability tests of eukaryotic cells to some simple chemical measurements in plasma treated liquid, the DIN SPEC can provide safety for users (investigators, patients, physicians), and accelerate technology transfer. In general, comprehensive knowledge of all formal reporting requirements in the respective country is needed to ensure that sufficient data management and proper documentation of procedures are performed to meet the respective regulatory standards.

A promising way of technology transfer in both industrial and biomedical applications of LTPs is the establishment of spin-off companies. The main advantage of this approach is that the required scientific understanding and detailed knowledge of the intended application is directly available from the inventors themselves. Obstacles here are often the lack of startup funding and, of course, the transition phase for any technological innovation. This does not exclude plasma technology. There is always the challenge to survive the *valley of death*, which is the transition from a laboratory prototype to a manufacturable and maintainable product.

Other issues encountered on the way to establishing new applications of plasma technology are visibility and economics. As a niche or merely supporting technology, it is often difficult to be perceived as an alternative to established technologies that have a large installed base. Resource consumption (e.g. energy, gas, electrode materials) and possible toxic byproducts (e.g. toxic species in the exhause such as fluorides) are barriers to success. If the goal is replacement of an existing technology, collaboration with interdisciplinary partners with knowledge of current production technologies and product development is beneficial. In all cases the specific regulatory, technological, infrastructural and operational conditions need to be considered. Life-cycle assessment processes and cyberphysical systems like digital twins will become more and more relevant [216]. It has to be demonstrated that the plasma technology has a positive impact on the complete process chain of the production engineering. Moreover, the advantages in the product life cycle including recycling has to be addressed. In summary, existing obstacles to technology transfer can be minimized by

- Developing standardized tests and operational procedures for industrial and medical plasma applications.
- Establishing data management and documentation standards that consider existing regulations and standard operating procedures.
- Expanding the development of standard and scalable plasma sources for various applications.
- Providing broad education about the benefits and risks of plasma technology in society.

Many of the topics discussed above (e.g. standard plasma sources, data and mechanisms, reporting, FAIR data principles) thus play key roles in efficient technology transfer.

## 11. Concluding remarks

The goals of transparency, reproducibility and collaboration in LTP research are both fundamental to the field and challenges to implement. The broad intellectual diversity of LTPs and their allied fields, the large variety of applications, and membership from the physical, biological and engineering sciences make rigidly applying a stringent set of standards difficult and probably not advisable. That said, working toward these goals while also embracing innovation should be a guiding principle for the field. This review has discussed several methods to achieve these goals, through proposed standards and best practices, while also acknowledging the role of judgement so that innovation is encouraged and not impeded.

The use of standard plasma sources such as the GECRC and the COST jet have improved our understanding of fundamental plasma processes. The use of standard plasma sources should be a tool toward improving fundamental understanding of plasma processes and speeding translation of scientific findings to applications, while not hindering innovation. Rather, they can act as ‘calibration sources’ to contextualize results, for diagnostics and modeling, and to facilitate new source development and scaled up processes. More community-driven designs for reference plasma sources and plasma-surface treatment systems are encouraged as they will likely promote fundamental understanding, benefiting the whole LTP community.

In contrast to other disciplines, recommended protocols for standard measurement techniques are less developed in plasma science, and for LTPs in particular. This situation is largely due to the challenging and strongly diverse nature of LTPs, often requiring diagnostic techniques specifically adapted to the distinct plasma environment and particular plasma parameters of interest. Key issues to consider in a proposed framework for diagnostic standards to support transparency, reliability and transferability of experimental measurements are: identifying and classifying the diagnostic technique, the regime of validity of the technique, analysis technique(s) or requirements for a range of plasma environments, the range of equipment and potential calibration procedures, and input fundamental data required for analysis of the measured data including uncertainty assessment.

The demand for improved and more widely applied plasma models will continue to drive the demand for data on key atomic and molecular processes. While sophisticated methods, both experimental and theoretical, will continue to provide important results for key processes, limitations of these methods both in terms of expense and capability will mean that other, cheaper and more approximate methods will continue to be used. Community based best practices for applying those approximate methods will work toward better comparisons of models. Machine learning is likely to provide a fruitful means of filling the many gaps in data provision for plasma modelling, a process that will also benefit from some standards or best practices.

Developing research software should consider the options of open-source and adopting FAIR principles early in the process. A possible roadmap involves planning; development, review and verification; sharing; and publishing. These practices could significantly contribute to elevating research standards, improving the quality of codes and computational predictions, reinforcing the conduit between codes and data (or databases), and promoting the transparent reporting on models and procedures.

Establishing the correctness of both computer codes and the mathematical models that they express has been a matter of increasing concern to many technical communities in recent years. Many LTP scientists share these concerns. However, the problems that arise are not simple. For many computational methods in wide use in the LTP community, canonical methods of demonstrating correctness (‘verification’) do not yet exist. Even when appropriate techniques are available, a comprehensive verification effort can consume significant resources that could be otherwise used. Model testing by comparison with experiments (‘validation’) may be even more time consuming and expensive. Consequently, authors, editorial boards, and other actors need to carefully assess what level of commitment to V&V activities is appropriate for any particular calculation. The answer may vary widely, depending on the nature of the calculation and the role of the calculation in the chain of scientific argument. For instance, in exploratory scientific research, a rather light V&V effort may be appropriate. In engineering prediction, the full machinery of formal V&V may be needed. Future progress likely requires both technical improvements and better understanding by the community of the broader issues that arise.

As scrutiny of scientific findings continues to grow among the general public as well as public institutions and governments, proper reporting of research findings in archival journal articles takes on an added urgency. The LTP community is fortunate that there has not been a high-profile retraction or reproducibility scandal in the recent past that diminishes public trust in the field and its findings. While journals and journal publishers have a role to play in establishing standard practices for data reporting, the LTP (and broader scientific) community at large, including authors, reviewers, editors, and those that use data, plays perhaps the most important role in setting expectations for proper data reporting.

The transfer from basic and applied research to new plasma-based technologies faces several challenges that differ for industrial plasma applications and the healthcare sector. While the former is primarily hampered by communication and upscaling issues, the latter is often constrained by the requirements of governmental approvals and device regulations. Wider adoption of standards and best practices for the development and application of plasma sources, diagnostic methods, plasma models and simulation codes, data storage, and reporting on all of these aspects would be of great benefit to technology transfer in both fields.

Perhaps the most challenging of standards is correlating plasma source operation with a plasma dose; a specified fluence of an excited state, ion or radical delivered by the plasma source. Ideally, the doses of reactivity delivered by plasma sources should be part of their specifications. We now generally lack this correlation and lack the ability to describe plasma sources in terms of the dose of individual reactive species delivered by the sources. As a result, applications are strongly coupled to a particular and sometimes unique plasma source. Overcoming this limitation, then opens the possibility of having interchangeable plasma sources, or using different plasmas sources to treat small surface areas and large surface areas. Defining doses and process requirements using dose might stimulate commercial development of a wide range of plasmas sources using the dose standard.

## Data Availability

The data used in preparation of this article appear in the article or in the cited references. Requests for additional data should be directed to the corresponding author.
